# 32nd International Austrian Winter Symposium: Zell am See, the Netherlands. 20-23 January 2016

**DOI:** 10.1186/s13550-016-0168-9

**Published:** 2016-04-18

**Authors:** W Langsteger, A Rezaee, W Loidl, HS Geinitz, F Fitz, M Steinmair, G Broinger, L Pallwien-Prettner, M Beheshti, L Imamovic, M Beheshti, G Rendl, D Hackl, O Tsybrovsky, M Steinmair, K Emmanuel, F Moinfar, C Pirich, W Langsteger, A Bytyqi, G Karanikas, M Mayerhöfer, O Koperek, B Niederle, M Hartenbach, T Beyer, K Herrmann, J Czernin, I Rausch, P Rust, MD DiFranco, M Lassen, A Stadlbauer, ME Mayerhöfer, M Hartenbach, M Hacker, T Beyer, K Binzel, R Magnussen, W Wei, MU Knopp, DC Flanigan, C Kaeding, MV Knopp, A Leisser, M Nejabat, M Hartenbach, G Kramer, M Krainer, M Hacker, A Haug, Wencke Lehnert, Karl Schmidt, Sharok Kimiaei, Marcus Bronzel, Andreas Kluge, CL Wright, K Binzel, J Zhang, Evan Wuthrick, Piotr Maniawski, MV Knopp, M Blaickner, E Rados, A Huber, M Dulovits, H Kulkarni, S Wiessalla, C Schuchardt, RP Baum, B Knäusl, D Georg, M Bauer, B Wulkersdorfer, W Wadsak, C Philippe, H Haslacher, M Zeitlinger, O Langer, M Bauer, M Feldmann, R Karch, W Wadsak, M Zeitlinger, MJ Koepp, M-C Asselin, E Pataraia, O Langer, M Zeilinger, C Philippe, M Dumanic, F Pichler, J Pilz, M Hacker, W Wadsak, M Mitterhauser, L Nics, B Steiner, M Hacker, M Mitterhauser, W Wadsak, A Traxl, Thomas Wanek, Kushtrim Kryeziu, Severin Mairinger, Johann Stanek, Walter Berger, Claudia Kuntner, Oliver Langer, S Mairinger, T Wanek, A Traxl, M Krohn, J Stanek, T Filip, M Sauberer, C Kuntner, J Pahnke, O Langer, D Svatunek, C Denk, M Wilkovitsch, T Wanek, T Filip, C Kuntner-Hannes, J Fröhlich, H Mikula, C Denk, D Svatunek, T Wanek, S Mairinger, J Stanek, T Filip, J Fröhlich, H Mikula, C Kuntner-Hannes, T Balber, J Singer, J Fazekas, C Rami-Mark, N Berroterán-Infante, E Jensen-Jarolim, W Wadsak, M Hacker, H Viernstein, M Mitterhauser, C Denk, D Svatunek, B Sohr, H Mikula, J Fröhlich, T Wanek, C Kuntner-Hannes, T Filip, S Pfaff, C Philippe, M Mitterhauser, M Hartenbach, M Hacker, W Wadsak, T Wanek, E Halilbasic, M Visentin, S Mairinger, B Stieger, C Kuntner, M Trauner, O Langer, P Lam, M Aistleitner, R Eichinger, C Artner, H Eidherr, C Vraka, A Haug, M Mitterhauser, L Nics, M Hartenbach, M Hacker, W Wadsak, H Kvaternik, R Müller, D Hausberger, C Zink, RM Aigner, U Cossío, M Asensio, A Montes, S Akhtar, Y te Welscher, R van Nostrum, V Gómez-Vallejo, J Llop, F VandeVyver, T Barclay, N Lippens, M Troch, L Hehenwarter, B Egger, J Holzmannhofer, M Rodrigues-Radischat, C Pirich, N Pötsch, I Rausch, D Wilhelm, M Weber, J Furtner, G Karanikas, A Wöhrer, M Mitterhauser, M Hacker, T Traub-Weidinger, T Cassou-Mounat, S Balogova, V Nataf, M Calzada, V Huchet, K Kerrou, J-Y Devaux, M Mohty, L Garderet, J-N Talbot, S Stanzel, G Pregartner, T Schwarz, V Bjelic-Radisic, B Liegl-Atzwanger, R Aigner, S Stanzel, F Quehenberger, RM Aigner, A Koljević Marković, Milica Janković, V Miler Jerković, M Paskaš, G Pupić, R Džodić, D Popović, MC Fornito, D Familiari, P Koranda, H Polzerová, I Metelková, L Henzlová, R Formánek, E Buriánková, M Kamínek, WH Thomson, C Lewis, WH Thomson, J O’Brien, G James, A Notghi, H Huber, I Stelzmüller, R Wunn, M Mandl, F Fellner, B Lamprecht, M Gabriel, MC Fornito, G Leonardi, WH Thomson, J O’Brien, G James, J Hudzietzová, J Sabol, M Fülöp

**Affiliations:** PET-CT Center Linz, Department of Nuclear Medicine & Endocrinology, St Vincent’s Hospital, Linz, Austria; Prostate Cancer Center Linz, Department of Urology, St Vincent’s Hospital, Linz, Austria; Department of Radiation Oncology, St Vincent’s Hospital, Linz, Austria; Department of Radiology, St Vincent’s Hospital, Linz, Austria; PET – CT Center Linz & Department of Nuclear Medicine & Endocrinology, St Vincent’s Hospital, Linz, Austria; Department of Surgery, St Vincent’s Hospital, Linz, Austria; Department of Pathology, St Vincent’s Hospital, Linz, Austria; Department of Nuclear Medicine and Endocrinology, Paracelsus Private Medical University Salzburg, St Vincent’s Hospital, Linz, Austria; Medical University of Vienna, Division of Nuclear Medicine, Vienna, Austria; Medical University of Vienna, Division of General and Pediatric Radiology, Vienna, Austria; Medical University of Vienna, Institute of Pathology, Vienna, Austria; Medical University Vienna, Division of Surgical Endocrinology, Vienna, Austria; QIMP, CMPBME, Medical University of Vienna, ᅟ, Austria; Department of Nuclear Medicine, University of Würzburg, ᅟ, Germany; Department of Molecular and Medical Pharmacology, UCLA, ᅟ, USA; Center for Medical Physics and Biomedical Engineering, Medical University of Vienna, ᅟ, Austria; Department of Nutritional Sciences, University of Vienna, ᅟ, Austria; Division of General and Pediatric Radiology, Department of Biomedical Imaging and Image-guided Therapy, Medical University of Vienna, ᅟ, Austria; Division of Nuclear Medicine, Department of Biomedical Imaging and Image-guided Therapy, Medical University of Vienna, ᅟ, Austria; Wright Center of Innovation in Biomedical Imaging, The Ohio State University, Columbus, OH USA; Sports Medicine, The Ohio State University, Columbus, OH USA; Sports Medicine, Pepperdine University, Malibu, CA USA; Division of Nuclear Medicine, Medical University of Vienna, ᅟ, Austria; ABX-CRO advanced pharmaceutical services (Forschungsgesellschaft mbH), Dresden, Germany; Wright Center of Innovation in Biomedical Imaging, The Ohio State University, Columbus, OH USA; Radiation Oncology, Wexner Medical Center at The Ohio State University, Columbus, OH USA; Clinical Science - Nuclear Medicine, Philips Healthcare, Cleveland, OH USA; AIT Austrian Institute of Technology, Health & Environment Department -Biomedical Systems, Vienna, Austria; Woogieworks Animation Studio, Perchtoldsdorf, Austria; THERANOSTICS Center for Molecular Radiotherapy and Molecular Imaging (PET/CT) ENETS Center of Excellence, Zentralklinik Bad Berka, ᅟ, Germany; Department of Radiation Oncology, Division of Medical Radiation Physics, Christian Doppler Laboratory for Medical Radiation Research for Radiation Oncology, Medical University of Vienna, ᅟ, Austria; Department of Clinical Pharmacology, Medical University of Vienna, Vienna, Austria; Department of Biomedical Imaging and Image-guided Therapy, Medical University of Vienna, Vienna, Austria; Department of Laboratory Medicine, Medical University of Vienna, Vienna, Austria; Health and Environment Department, AIT Austrian Institute of Technology GmbH, Seibersdorf, Austria; Department of Clinical Pharmacology, Medical University of Vienna, Vienna, Austria; Wolfson Molecular Imaging Centre, University of Manchester, Manchester, UK; University College London, London, UK; Center for Medical Statistics, Informatics, and Intelligent Systems, Medical University of Vienna, Vienna, Austria; Department of Biomedical Imaging and Image-guided Therapy, Medical University of Vienna, Vienna, Austria; Department of Neurology, Medical University of Vienna, Vienna, Austria; Department of Biomedical Imaging and Image-guided Therapy, Division of Nuclear Medicine, Radiopharmacy and Experimental Nuclear Medicine, Medical University of Vienna, ᅟ, Austria; Ludwig Boltzmann Institute for Applied Diagnostics, Vienna, Austria; Department of Biomedical Imaging and Image guided Therapy, Division of Nuclear Medicine, Medical University of Vienna, ᅟ, Austria; Ludwig Boltzmann Institute for Applied Diagnostics, Vienna, Austria; Biomedical Systems, Health & Environment Department, AIT Austrian Institute of Technology GmbH, Seibersdorf, Austria; Institute of Cancer Research, Department of Medicine I, Medical University of Vienna, Vienna, Austria; Department of Clinical Pharmacology, Medical University of Vienna, Vienna, Austria; Health & Environment Department, AIT Austrian Institute of Technology GmbH, Seibersdorf, Austria; Department of Neuro-/Pathology, University of Oslo (UiO) and Oslo University Hospital (OUS), Oslo, Norway; Department of Clinical Pharmacology, Medical University of Vienna, Vienna, Austria; Institute of Applied Synthetic Chemistry, TU Wien, Vienna, Austria; Austrian Institute of Technology, Vienna, Austria; Institute of Applied Synthetic Chemistry, TU Wien, Vienna, Austria; Austrian Institute of Technology, Biomedical Systems, Vienna, Austria; Biomedical Imaging and Image-Guided Therapy, Division of Nuclear Medicine, Medical University of Vienna, ᅟ, Austria; Department of Immunology and Oncology, Institute of Pathophysiology and Allergy Research, Center of Pathophysiology, Infectiology and Immunology, Medical University of Vienna, ᅟ, Austria; Department of Comparative Medicine, Messerli Research Institute of the University of Veterinary Medicine Vienna, Medical University Vienna and University Vienna, ᅟ, Austria; Department of Pharmaceutical Technology and Biopharmaceutics, Faculty of Life Sciences, University of Vienna, ᅟ, Austria; Institute of Applied Synthetic Chemistry, TU Wien, Vienna, Austria; Austrian Institute of Technology, Biomedical Systems, Vienna, Austria; Department of Biomedical Imaging and Image-guided Therapy, Division of Nuclear Medicine, Medical University of Vienna, ᅟ, Austria; Department of Inorganic Chemistry, University of Vienna, ᅟ, Austria; LBI for Applied Diagnostics, Vienna, Austria; Health and Environment Department, Biomedical Systems, AIT Austrian Institute of Technology GmbH, Seibersdorf, Austria; Division of Gastroenterology and Hepatology, Department of Internal Medicine III, Medical University of Vienna, Vienna, Austria; Department of Clinical Pharmacology and Toxicology, University Hospital, Zurich, Switzerland; Department of Clinical Pharmacology, Medical University of Vienna, ᅟ, Austria; IASON GmbH, Feldkirchnerstraße 4, A-8054 Graz-Seiersberg, Austria; Department of Biomedical Imaging and Image-guided Therapy, Division of Nuclear Medicine, Medical University of Vienna, ᅟ, Austria; Department of Nutritional Sciences, Faculty of Life Sciences, University of Vienna, ᅟ, Austria; LBI for Applied Diagnostics, Vienna, Austria; Department of Radiology, Division of Nuclear Medicine, Medical University of Graz, ᅟ, Austria; Seibersdorf Labor GmbH, ᅟ, Austria; CIC biomaGUNE, Edificio Empresarial “C”, Paseo de Miramón 182, 20009 Donostia, Spain; Engineering Department, Ingeniatrics Tecnologies, P.I. Parque Plata, Camino Mozárabe 41, 41900 Camas-Sevilla, Spain; Department of Pharmaceutics, University of Utrecht, Utrecht, The Netherlands; AZ St-Lucas Gent, ᅟ, Belgium; Department of Nuclear Medicine and Endocrinology, University Hospital Salzburg, Paracelsus Private Medical University Salzburg, ᅟ, Germany; Department of Biomedical Imaging and Image-guided Therapy, Medical University of Vienna, Vienna, Austria; Center for Medical Physics and Biomedical Engineering, Medical University of Vienna, Vienna, Austria; Institute of Neurology, Medical University of Vienna, Vienna, Austria; Department of Nuclear Medicine, Hôpital Saint Antoine, AP-HP et Université Pierre et Marie Curie (UPMC), Paris, France; Department of Nuclear Medicine, Hôpital Tenon, AP-HP & Université Pierre et Marie Curie (UPMC), Paris, France; Department of Nuclear Medicine, Comenius university & St. Elisabeth Oncology Institute, Bratislava, Slovakia; Radiopharmacy, Hôpital Tenon, AP-HP, Paris, France; Hematology, Université Pierre et Marie Curie, Paris, France; Hôpital Saint-Antoine, AP-HP, Paris, France; INSERM UMRs U938, Paris, France; Medical University of Graz, Department of Radiology, Division of Nuclear Medicine, ᅟ, Austria; Medical University of Graz, Institute for Medical Informatics, Statistics and Documentation, ᅟ, Austria; Medical University of Graz, Department of Gynecology and Obstetrics, ᅟ, Austria; Medical University of Graz, Institute of Pathology, ᅟ, Austria; Medical University of Graz, Department of Radiology, Division of Nuclear Medicine, ᅟ, Austria; Institute for Medical Informatics, Statistics, and Documentation, ᅟ, Austria; Institute of Oncology and Radiology of Serbia, Pasterova 14, 11000 Belgrade, Serbia; National Cancer Research Center Serbia, University of Belgrade- School of Electrical Engineering, ᅟ, Serbia; National Cancer Research Center Serbia, Innovation Center, University of Belgrade – Faculty of Electrical Engineering, ᅟ, Serbia; Nuclear Medicine Department and PET/CT center – A.R.N.A.S “ Garibaldi – Nesima”, Via Palermo 636, 95122 Catania, Italy; Department of Nuclear Medicine, Palacky University and University Hospital, Olomouc, Czech Republic; Physics and Nuclear Medicine Department City Hospital, Birmingham, UK; Maternity Department City Hospital, Birmingham, UK; Physics and Nuclear Medicine Department, City Hospital, Birmingham, UK; Institut für Nuklearmedizin und Endokrinologie, AKH Linz/Kepler Universitätsklinikum, ᅟ, Austria; Abteilung für Lungenkrankheiten, AKH Linz/Kepler Universitätsklinikum, ᅟ, Austria; Zentrales Radiologie-Institut, AKH Linz/Kepler Universitätsklinikum, ᅟ, Austria; Nuclear Medicine Department and PET/CT center – A.R.N.A.S “ Garibaldi – Nesima”, Via Palermo 636, 95122 Catania, Italy; Heart-Failure Department – Azienda Ospedaliera Universitaria “Policlinico- Vittorio Emanuele”, Catania, Italy; Physics and Nuclear Medicine Department, City Hospital, Birmingham, UK; Faculty of Biomedical Engineering, CTU, Prague, Czech Republic; Faculty of Safety Management, PACR, Prague, Czech Republic; Faculty of Public Health, SMU, Bratislava, Slovak Republic

## Abstract

A1 68Ga-PSMA PET/CT in staging and restaging of Prostate Cancer Patients: comparative study with 18F-Choline PET/CT

W Langsteger, A Rezaee, W Loidl, HS Geinitz, F Fitz, M Steinmair, G Broinger, L Pallwien-Prettner, M Beheshti

A2 F18 Choline PET – CT: an accurate diagnostic tool for the detection of parathyroid adenoma?

L Imamovic, M Beheshti, G Rendl, D Hackl, O Tsybrovsky, M Steinmair, K Emmanuel, F Moinfar, C Pirich, W Langsteger

A3 [18F]Fluoro-DOPA-PET/CT in the primary diagnosis of medullary thyroid carcinoma

A Bytyqi, G Karanikas, M Mayerhöfer, O Koperek, B Niederle, M Hartenbach

A4 Variations of clinical PET/MR operations: An international survey on the clinical utilization of PET/MRI

T Beyer, K Herrmann, J Czernin

A5 Standard Dixon-based attenuation correction in combined PET/MRI: Reproducibility and the possibility of Lean body mass estimation

I Rausch, P Rust, MD DiFranco, M Lassen, A Stadlbauer, ME Mayerhöfer, M Hartenbach, M Hacker, T Beyer

A6 High resolution digital FDG PET/MRI imaging for assessment of ACL graft viability

K Binzel, R Magnussen, W Wei, MU Knopp, DC Flanigan, C Kaeding, MV Knopp

A7 Using pre-existing hematotoxicity as predictor for severe side effects and number of treatment cycles of Xofigo therapy

A Leisser, M Nejabat, M Hartenbach, G Kramer, M Krainer, M Hacker, A Haug

A8 QDOSE – comprehensive software solution for internal dose assessment

Wencke Lehnert, Karl Schmidt, Sharok Kimiaei, Marcus Bronzel, Andreas Kluge

A9 Clinical impact of Time-of-Flight on next-generation digital PET imaging of Yttrium-90 radioactivity following liver radioembolization

CL Wright, K Binzel, J Zhang, Evan Wuthrick, Piotr Maniawski, MV Knopp

A10 Snakes in patients! Lessons learned from programming active contours for automated organ segmentation

M Blaickner, E Rados, A Huber, M Dulovits, H Kulkarni, S Wiessalla, C Schuchardt, RP Baum, B Knäusl, D Georg

A11 Influence of a genetic polymorphism on brain uptake of the dual ABCB1/ABCG2 substrate [11C]tariquidar

M Bauer, B Wulkersdorfer, W Wadsak, C Philippe, H Haslacher, M Zeitlinger, O Langer

A12 Outcome prediction of temporal lobe epilepsy surgery from P-glycoprotein activity. Pooled analysis of (R)-[11C]-verapamil PET data from two European centres

M Bauer, M Feldmann, R Karch, W Wadsak, M Zeitlinger, MJ Koepp, M-C Asselin, E Pataraia, O Langer

A13 In-vitro and in-vivo characterization of [18F]FE@SNAP and derivatives for the visualization of the melanin concentrating hormone receptor 1

M Zeilinger, C Philippe, M Dumanic, F Pichler, J Pilz, M Hacker, W Wadsak, M Mitterhauser

A14 Reducing time in quality control leads to higher specific radioactivity of short-lived radiotracers

L Nics, B Steiner, M Hacker, M Mitterhauser, W Wadsak

A15 In vitro 11C-erlotinib binding experiments in cancer cell lines with epidermal growth factor receptor mutations

A Traxl, Thomas Wanek, Kushtrim Kryeziu, Severin Mairinger, Johann Stanek, Walter Berger, Claudia Kuntner, Oliver Langer

A16 7-[11C]methyl-6-bromopurine, a PET tracer to measure brain Mrp1 function: radiosynthesis and first PET evaluation in mice

S Mairinger, T Wanek, A Traxl, M Krohn, J Stanek, T Filip, M Sauberer, C Kuntner, J Pahnke, O Langer

A17 18F labeled azidoglucose derivatives as “click” agents for pretargeted PET imaging

D Svatunek, C Denk, M Wilkovitsch, T Wanek, T Filip, C Kuntner-Hannes, J Fröhlich, H Mikula

A18 Bioorthogonal tools for PET imaging: development of radiolabeled 1,2,4,5-Tetrazines

C Denk, D Svatunek, T Wanek, S Mairinger, J Stanek, T Filip, J Fröhlich, H Mikula, C Kuntner-Hannes

A19 Preclinical evaluation of [18F]FE@SUPPY- a new PET-tracer for oncology

T Balber, J Singer, J Fazekas, C Rami-Mark, N Berroterán-Infante, E Jensen-Jarolim, W Wadsak, M Hacker, H Viernstein, M Mitterhauser

A20 Investigation of Small [18F]-Fluoroalkylazides for Rapid Radiolabeling and In Vivo Click Chemistry

C Denk, D Svatunek, B Sohr, H Mikula, J Fröhlich, T Wanek, C Kuntner-Hannes, T Filip

A21 Microfluidic 68Ga-radiolabeling of PSMA-HBED-CC using a flow-through reactor

S Pfaff, C Philippe, M Mitterhauser, M Hartenbach, M Hacker, W Wadsak

A22 Influence of 24-nor-ursodeoxycholic acid on hepatic disposition of [18F]ciprofloxacin measured with positron emission tomography

T Wanek, E Halilbasic, M Visentin, S Mairinger, B Stieger, C Kuntner, M Trauner, O Langer

A23 Automated 18F-flumazenil production using chemically resistant disposable cassettes

P Lam, M Aistleitner, R Eichinger, C Artner

A24 Similarities and differences in the synthesis and quality control of 177Lu-DOTA-TATE, 177Lu -HA-DOTA-TATE and 177Lu-DOTA-PSMA (PSMA-617)

H Eidherr, C Vraka, A Haug, M Mitterhauser, L Nics, M Hartenbach, M Hacker, W Wadsak

A25 68Ga- and 177Lu-labelling of PSMA-617

H Kvaternik, R Müller, D Hausberger, C Zink, RM Aigner

A26 Radiolabelling of liposomes with 67Ga and biodistribution studies after administration by an aerosol inhalation system

U Cossío, M Asensio, A Montes, S Akhtar, Y te Welscher, R van Nostrum, V Gómez-Vallejo, J Llop

A27 Fully automated quantification of DaTscan SPECT: Integration of age and gender differences

F VandeVyver, T Barclay, N Lippens, M Troch

A28 Lesion-to-background ratio in co-registered 18F-FET PET/MR imaging – is it a valuable tool to differentiate between low grade and high grade brain tumor?

L Hehenwarter, B Egger, J Holzmannhofer, M Rodrigues-Radischat, C Pirich

A29 [11C]-methionine PET in gliomas - a retrospective data analysis of 166 patients

N Pötsch, I Rausch, D Wilhelm, M Weber, J Furtner, G Karanikas, A Wöhrer, M Mitterhauser, M Hacker, T Traub-Weidinger

A30 18F-Fluorocholine versus 18F-Fluorodeoxyglucose for PET/CT imaging in patients with relapsed or progressive multiple myeloma: a pilot study

T Cassou-Mounat, S Balogova, V Nataf, M Calzada, V Huchet, K Kerrou, J-Y Devaux, M Mohty, L Garderet, J-N Talbot

A31 Prognostic benefit of additional SPECT/CT in sentinel lymph node mapping of breast cancer patients

S Stanzel, G Pregartner, T Schwarz, V Bjelic-Radisic, B Liegl-Atzwanger, R Aigner

A32 Evaluation of diagnostic value of TOF-18F-FDG PET/CT in patients with suspected pancreatic cancer

S Stanzel, F Quehenberger, RM Aigner

A33 New quantification method for diagnosis of primary hyperpatahyroidism lesions and differential diagnosis vs thyropid nodular disease in dynamic scintigraphy

A Koljević Marković, Milica Janković, V Miler Jerković, M Paskaš, G Pupić, R Džodić, D Popović

A34 A rare case of diffuse pancreatic involvement in patient with merkel cell carcinoma detected by 18F-FDG

MC Fornito, D Familiari

A35 TSH-stimulated 18F-FDG PET/CT in the diagnosis of recurrent/metastatic radioiodine-negative differentiated thyroid carcinomas in patients with various thyroglobuline levels

P Koranda, H Polzerová, I Metelková, L Henzlová, R Formánek, E Buriánková, M Kamínek

A36 Breast Dose from lactation following I131 treatment

WH Thomson, C Lewis

A37 A new concept for performing SeHCAT studies with the gamma camera

WH Thomson, J O’Brien, G James, A Notghi

A38 Whole body F-18-FDG-PET and tuberculosis: sensitivity compared to x-ray-CT

H Huber, I Stelzmüller, R Wunn, M Mandl, F Fellner, B Lamprecht, M Gabriel

A39 Emerging role 18F-FDG PET-CT in the diagnosis and follow-up of the infection in heartware ventricular assist system (HVAD)

MC Fornito, G Leonardi

A40 Validation of Poisson resampling software

WH Thomson, J O’Brien, G James

A41 Protection of PET nuclear medicine personnel: problems in satisfying dose limit requirements

J Hudzietzová, J Sabol, M Fülöp

## A1 68Ga-PSMA PET/CT in staging and restaging of Prostate Cancer Patients: comparative study with 18F-Choline PET/CT

### W Langsteger^1^, A Rezaee^1^, W Loidl^2^, HS Geinitz^3^, F Fitz^1^, M Steinmair^1^, G Broinger^4^, L Pallwien-Prettner^5^, M Beheshti^1^

#### ^1^PET-CT Center Linz, Department of Nuclear Medicine & Endocrinology, St Vincent’s Hospital, Linz, Austria; ^2^Prostate Cancer Center Linz, Department of Urology, St Vincent’s Hospital, Linz, Austria; ^3^Department of Radiation Oncology, St Vincent’s Hospital, Linz, Austria; ^4^Department of Radiology, St Vincent’s Hospital, Linz, Austria

**Aim:** 11C- and 18F-choline PET/CT have been established as a promising modality in the assessment of prostate cancer patients1. However, it suffers to detect small malignant lesions. 68Ga-PSMA (PSMA) PET/CT showed promising in the detection of small lesions with a high tumor to background contrast2.

This study was designed for comparison of detection rate between PSMA and 18F-fluoromethylcholine (FCH) PET/CT scan in pre or post-op prostate cancer patients.

**Methods:** In this prospective study 15 consecutive prostate cancer patients (mean age 67.9, range 57–83) underwent both PSMA and FCH PET/CT with a maximum interval of 4 weeks without any treatment in between. The imaging modalities were performed in 9 patients (mean age: 70.3; range: 63–83) in pre- and 6 Patients (mean age: 64.2; range: 57–71) in post-operative setting.

Prostate cancer patients with histopathologic verification or biochemical recurrence were included in this study. Patients with systemic therapy and known second cancer were excluded. Pathologic findings in each imaging modalities have to be clarified histopathologically or by conventional imaging modalities and/or clinical follow-up.

**Results:** Staging: The mean of PSA was 35.1 ng/ml (range: 3.44 - 81.17 ng/ml). Pathologically increased tracer uptake was detected on both imaging modalities in the prostate gland in all patients [mean size on PSMA PET/CT: 20 mm (range: 12–43); FCH PET/CT: 23.6 mm (range: 12–34) & mean SUVmax on PSMA PET/CT: 16.2 (range: 6.1-28.1); FCH PET/CT: 7.4 (range: 3.6-15.9)].

Overall, a total number of 15 and 14 positive lymph nodes were detected on PSMA PET/CT and FCH PET/CT images, respectively. Malignant lymph nodes showed significantly higher uptake on PSMA- comparing FCH PET/CT [mean SUVmax on PSMA PET/CT: 12.8 (range: 2.8-34); FCH PET/CT: 6.6 (range: 2.3-11.9)]. However, there was no appreciable difference in the tracer intensity of the detected bony lesions in each modality.

Restaging: The mean of PSA was 2.3 ng/ml (range: 0.48 - 5.35). Local recurrence with pathologically increased tracer uptake was detected in one patient on both imaging modalities, However, it shows markedly higher uptake on PSMA PET/CT (SUVmax: PSMA: 12.4; FCH: 4.9).

In addition, PSMA PET/CT was able to detect higher number of metastatic lymph nodes compared with FCH PET/CT (14 vs. 10) with significantly better tumor to background ratio (SUVmax 8.7 on PSMA vs 4.45 on FCH).

One false positive bone lesion was detected on PSMA PET/CT study. Although the MRI was negative, follow-up imaging was planned within 4–6 months. Also, two false positive bone lesions were detected on FCH PET/CT images in bilateral femurs. Moreover, PSMA PET/CT was false negative in one bony lesion detected by FCH PET/CT.

**Conclusion:** PSMA PET/CT reveals a more promising role for staging and re-staging of prostate cancer patients even with low PSA level. Markedly higher tumor to background contrast is seen on PSMA PET/CT which allows higher detection rate especially in the small lesions. However, the value of this modality in the assessment of bone metastases should be further evaluated in future studies.

**References**

1. Mohsen Beheshti et al. Impact of 18F-Choline PET/CT in Prostate Cancer Patients with Biochemical Recurrence: Influence of Androgen Deprivation Therapy and Correlation with PSA Kinetics. J Nucl Med 2013; 54:1–8

2. Joshua Morigi et al. Prospective Comparison of 18F-Fluoromethylcholine Versus 68Ga-PSMA PET/CT in Prostate Cancer Patients Who Have Rising PSA After Curative Treatment and Are Being Considered for Targeted Therapy. J Nucl Med 2015; 56:1185–1190

## A2 F18 Choline PET – CT: an accurate diagnostic tool for the detection of parathyroid adenoma?

### L Imamovic^1^, M Beheshti^1^, G Rendl^4^, D Hackl^2^, O Tsybrovsky^3^, M Steinmair^1^, K Emmanuel^2^, F Moinfar^3^, C Pirich^4^, W Langsteger^1^

#### ^1^PET – CT Center Linz & Department of Nuclear Medicine & Endocrinology, St Vincent’s Hospital, Linz, Austria; ^2^Department of Surgery, St Vincent’s Hospital, Linz, Austria; ^3^Department of Pathology, St Vincent’s Hospital, Linz, Austria; ^4^Department of Nuclear Medicine and Endocrinology, Paracelsus Private Medical University Salzburg, St Vincent’s Hospital, Linz, Austria

**Aim:** In this prospective multicenter study we assessed the value of 18F-Choline (FCH) PET – CT comparing 99mTc-Sestamibi SPECT-CT in the detection of parathyroid adenoma in patients with primary hyperparathyroidism.

**Methods:** Both 99mTc-Sestamibi SPECT-CT and FCH PET – CT were performed in 74 consecutive patients with biochemical evidence of primary hyperparathyroidism. At least one abnormal 99mTc-Sestamibi and/or FCH focus corresponding to a parathyroid gland or ectopic parathyroid tissue was considered as positive finding. Surgical exploration and resection was performed in 34 patients with positive findings in at least one imaging modality. The results of imaging modalities were verified with histopathologic findings as the standard of truth.

**Results:** In patients who underwent surgery, mean serum calcium and parathormone (PTH) level was 2.76 ± 0.32 mmol/l and 176.93 ± 165.30 pg/ml, respectively. Intraoperative PTH monitoring showed a significant drop in PTH level in all patients. In a patient-based analysis, FCH PET – CT was able to detect parathyroid adenoma in 97 % of patients (33/34) while 99mTC-Sestamibi SPECT-CT was positive in 62 % of patients (21/34). The mean serum calcium and PTH levels of patients with negative imaging modalities were 2.41 ± 0.24 mmol/l and 104.87 ± 41.33 pg/ml, respectively. The size and weight of parathyroid adenomas in patients with only FCH PET – CT positive imaging were 14.1 mm and 1.5 g, respectively. Both imaging modalities were negative in 8 patients with evidence of primary hyperparathyroidism.

**Conclusion:** In this prospective multicenter study, FCH PET – CT showed promising results - clearly superior to 99mTC-Sestamibi SPECT-CT - in the localization of parathyroid adenoma in patients with primary hyperparathyroidism.

## A3 [18F]Fluoro-DOPA-PET/CT in the primary diagnosis of medullary thyroid carcinoma

### A Bytyqi^1^, G Karanikas^1^, M Mayerhöfer^2^, O Koperek^3^, B Niederle^4^, M Hartenbach^1^

#### ^1^Medical University of Vienna, Division of Nuclear Medicine, Vienna, Austria; ^2^Medical University of Vienna, Division of General and Pediatric Radiology, Vienna, Austria; ^3^Medical University of Vienna, Institute of Pathology, Vienna, Austria; ^4^Medical University Vienna, Division of Surgical Endocrinology, Vienna, Austria

**Aim:** Evaluation of [18F]Fluoro-DOPA-PET/CT in patients with primary medullary thyroid carcinoma (MTC) prior to total thyroidectomy and lymph node dissection.

**Methods:** 33 patients with elevated basal calcitonin levels and pathological peak stimulated calcitonin were examined with [18F]Fluoro-DOPA-PET/CT prior to surgery. The post-operative histological findings served as standard of reference for tumor- and lymph node detection. Post-operative basal und stimulated calcitonin levels as well as follow-up calcitonin were documented and correlated with pre-surgical parameters (DOPA-PET SUV, PET/CT staging, bas./stim. calcitonin) by means of ROC-Analysis and contingency tables.

**Results:** In all patients, primary MTC was histologically verified post-surgery. 29 out of 33 patients showed increased DOPA-Decarboxylase activity in the primary tumor (Sens.: 88 %; mean SUVmax: 10,3). Undetected tumors were exclusively staged pT1a. Lymph node staging showed a sens./spec./acc./ppv/npv of 65 %/100 %/79 %/100 %/65 % (P < 0,001). Pre-surgical cN1b-status in [18F]Fluoro-DOPA-PET/CT was predictive for postsurgical persistently elevated calcitonin levels (sens. 85 %, spec. 90 %, ppv 85 %; P < 0.001), as well as pre-surgical basal calcitonin levels with an AUC of 0.84 (P = 0.009). SUV-parameters did not show any significance in that matter.

**Conclusion:** [18F]Fluoro-DOPA-PET/CT detected increased DOPA-Decarboxylase activity in almost all primary MTCs, with the exception of few pT1a-tumors. Evaluation of lymph node status is highly specific and has a high positive predictive value, whereas a cN1b-stage as well as highly elevated basal calcitonin levels are predictive for post-surgical tumor persistency.

## A4 Variations of clinical PET/MR operations: An international survey on the clinical utilization of PET/MRI

### T Beyer^1^, K Herrmann^2,3^, J Czernin^3^

#### ^1^QIMP, CMPBME, Medical University of Vienna, Austria; ^2^Department of Nuclear Medicine, University of Würzburg, Würzburg, Germany; ^3^Department of Molecular and Medical Pharmacology, UCLA, USA

**Aim:** This survey aims at gathering information about PET/MR operations worldwide to determine the current clinical applications of PET/MR imaging.

**Methods:** An internet-based survey of active PET/MR users was ran between 05/2015 and 08/2015 using surveymonkey. All 69 active PET/MR sites were invited to partake in this survey. The survey was composed of 37 questions related to (A) PET/MR center and installation, (B) variations in imaging protocols and (C) potential future applications.

**Results:** Responses were collected from 38 (55 %) sites: North America (24 %), Europe (59 %) and APAC (17 %) corresponding to the regional installations worldwide. Sites have operated PET/MR for (27 ± 16) months with 58 % sites having 2+ years experience.

Sites used PET/MR for indications in oncology (66 %), neurology (18 %), other (10 %) and cardiology (6 %). The most frequent indications in oncology are brain, prostate, gastrointestinal and head/neck cancer. Tracers used most frequently for neuro-PET/MRI are [18F]FDG, [18F]FET and amyloid imaging compounds. Likewise most frequently used travers in oncology are [18F]FDG, somatostatin receptor ligands and choline analogues. [18F]FDG was also most frequently used for other applications.

Users consider oncology with a focus on prostate and head/neck cancer a key application of PET/MR, as well as and neurology/neurodegenerative imaging. Pediatric imaging was named by one site only. Responses to upcoming key applications were mixed, thereby representing essentially the same variety of indications that PET/MR is used for today with the exception that many sites anticipate more frequent use of PET/MR in cardiac patients, and in patients with cancers of soft tissues, prostate and gynecological cancers.

**Conclusion:** An international survey among early adopters of PET/MR reveals a mix of clinical routine and research applications with a focus on oncology and neurology. The future of PET/MRI is seen in expanded oncology and cardiac applications whereby system cost and the ability to fully integrate MR and PET information are considered key promotional factors.

## A5 Standard Dixon-based attenuation correction in combined PET/MRI: Reproducibility and the possibility of Lean body mass estimation

### I Rausch^1^, P Rust^2^, MD DiFranco^1^, M Lassen^1^, A Stadlbauer^3^, ME Mayerhöfer^3^, M Hartenbach^4^, M Hacker^4^ and T Beyer^1^

#### ^1^Center for Medical Physics and Biomedical Engineering, Medical University of Vienna, Vienna, Austria; ^2^Department of Nutritional Sciences, University of Vienna, Vienna, Austria; ^3^Division of General and Pediatric Radiology, Department of Biomedical Imaging and Image-guided Therapy, Medical University of Vienna, Austria; ^4^Division of Nuclear Medicine, Department of Biomedical Imaging and Image-guided Therapy, Medical University of Vienna, Vienna, Austria

**Aim:** To assess the reproducibility of standard, Dixon-based attenuation correction (MR-AC) in positron emission tomography/magnetic resonance (PET/MR) imaging and to evaluate the possibility of deriving a patient-specific lean body mass (LBM) from these MR-AC data for LBM based SUV quantification.

**Methods:** Ten subjects who underwent a body composition measurement using air displacement plethysmography (ADP) where included in this study. Subjects were positioned in a fully-integrated PET/MR system and three consecutive multi-bed acquisitions of the standard MR-AC image data were acquired. For each subject and MR-AC map, the following compartmental volumes were calculated: total-body (TB), soft tissue (ST), fat (F) and intermediate tissue (IT). Intra-subject differences in TB and sub-compartmental volumes (ST, F, L and IM) were assessed by means of coefficients of variation (CV) for all MR-AC maps and excluding those with major artifacts.

ADP measurements were used to calculate a reference LBM (LBM(ADP)). A second LBM estimate was derived from available MR-AC data: LBM(MR-AC) = (V(ST)*ρ(ST) + V(IM)*ρ(IM)*0.5)/(V(ST)*ρ(ST) + V(F)*ρ(F) + V(IM)*ρ(IM))*BW, where V is the respective tissue volume, ρ is the tissue-dependent density and BW is the body weight. An additional LBM estimate was obtained from a gender-specific formula (LBM(Formula)). Pearson’s correlation was calculated for LBM(ADP), LBM(MR-AC) and LBM(Formula). Further, linear regression analysis was performed on LBM(MR-AC) and LBM(ADP).

**Results:** Mean CV of the TB volume for all 30 scans was (2.1 ± 1.9)%. When excluding missing tissue artifacts, the CV was reduced to (0.3 ± 0.2)%. Mean CV for the sub-compartments before and after excluding artifacts was ST: (0.9 ± 1.1)% and (0.7 ± 0.7)%, F: (2.9 ± 4.1)% and (1.3 ± 1.0)%, and IM: (3.6 ± 3.7)% and (1.3 ± 0.7)%, respectively.

Correlation was highest for LBM(MR-AC) and LBM(ADP) (r = 0.99). Linear regression of data excluding artifacts resulted in a scaling factor of 1.06 for LBM(MR-AC).

**Conclusion:** LBM estimation from DIXON MR-AC maps correlates well with standard LBM and thus offers routine SUV(LBM)-based quantification in PET/MR. However, MR-AC images must be controlled for systematic artifacts, like missing tissue and tissue swaps, which limit MR-AC reproducibility.

## A6 High resolution digital FDG PET/MRI imaging for assessment of ACL graft viability

### K Binzel^1^, R Magnussen^2^, W Wei^1^, MU Knopp^3^, DC Flanigan^2^, C Kaeding^2^, MV Knopp^1^

#### ^1^Wright Center of Innovation in Biomedical Imaging, The Ohio State University, Columbus, OH, USA; ^2^Sports Medicine, The Ohio State University, Columbus OH, USA; ^3^Sports Medicine, Pepperdine University, Malibu, CA, USA

**Aim:** Injury to the anterior cruciate ligament (ACL) is common, particularly among young athletes. Reconstruction with a graft helps restore stability and function to the knee, as the graft heals through the process of ligamentization1. Anatomic imaging alone cannot provide insight as to the progress of graft ligamentization, however. Combined 18F-FDG PET/MR imaging on current systems has been shown to be feasible for the evaluation of ACL graft viability following reconstructive surgery. We evaluated the capabilities of high resolution PET/MRI through combined use of a next generation digital photon counting PET/CT and 3 T MRI in an on-going multi-modality imaging study for the assessment of graft viability.

**Methods:** 10 patients had a standard of care MRI on a 3 T Ingenia CX. Proton density and T1 weighted sequences were acquired on all three planes, with a 3D high resolution image acquired on the sagittal plane. Low-dose PET/CT acquisitions were performed on the Vereos digital PET/CT (all Philips Healthcare). A foam mold of the MR knee coil was used to match knee positioning between PET and MR acquisitions. A single bed position centered on the knees was acquired following a 111 MBq 18F-FDG injection. Listmode data were reconstructed using a 144x144 matrix with 4 mm3 voxel volumes, a 288x288 matrix with 2 mm3 volumes, and a 576x576 matrix with 1 mm3 voxel volumes. Each reconstruction matrix size also used the option of a Gaussian filter and point spread function correction. Patients were grouped according to time since surgery and SUVmax was measured in the proximal, middle, and distal portions of the graft, femoral and tibial tunnels, and the posterior cruciate ligament (PCL), and quadriceps muscle for reference. Matched ROIs were drawn in the contralateral healthy knee.

**Results:** In all cases, PET and MR images were readily co-registered for quantitative evaluation. Excellent image quality was seen at all reconstruction matrix sizes, with the smaller voxel volumes improving visual evaluation of the heterogeneity of uptake throughout the evaluated ACL grafts. As previously validated in phantom tests, the use of smaller voxel volumes also improved quantification of graft metabolic uptake. SUVs measured on 2 and 1 mm3 images were found to be greater than those of default 4 mm3 images for regions of interest in the graft and bone tunnels. SUVs in background regions were less affected by the change in reconstruction matrix size. Addition of the Gaussian filter and point spread function correction during reconstruction further improved visual and quantitative precision. As in previous evaluation of ACL grafts by conventional PET, the trend was seen whereby the metabolic activity in the graft and bone tunnels decreased with longer recovery times since surgery.

**Conclusion:** PET/MRI imaging of the knee, particularly ACL grafts, benefits from the improved signal to noise and capacity for higher resolution reconstruction facilitated by use of PET digital photon counting and digital MR coil systems. The primary advantage in digital PET imaging of small structures is due to the improved quantitative precision enabled by a reduction of partial volume effects. Higher matrix PET reconstruction also enables better voxel-wise registration to MRI. The improved accuracy of quantification with digital PET will allow for more detailed analysis and correlation with clinical evaluations of graft healing and rehabilitation planning following reconstructive surgery.

**References**

1. Janssen RP, Scheffler SU. Intra-articular remodelling of hamstring tendon grafts after anterior cruciate ligament reconstruction. Knee Surg Sports Traumatol Arthrosc. Sep 2014;22(9):2102–2108. PMID: 23982759.

## A7 Using pre-existing hematotoxicity as predictor for severe side effects and number of treatment cycles of Xofigo therapy

### A Leisser, M Nejabat, M Hartenbach, G Kramer, M Krainer, M Hacker, A Haug

#### Division of Nuclear Medicine, Medical University of Vienna, Vienna, Austria

**Aim:** Hematopoetic toxicity is regarded a major problem of treatment with Xofigo. In this study we analyzed whether pre-therapeutic hematotoxicity (HT) influences the occurrence of side-effects.

**Methods:** In 54 patients with metastatic CRPC that underwent Xofigo therapy data on hemoglobin-levels (Hb), number of platelets (Plts) and leukocytes (Leuk) before, during and after therapy were collected. Pre-therapeutic HT and adverse events (AE) were scored (grade 0–4) according to the CTCAE recommendations. For further analysis patients were categorized with regard to their initial HT grade and correlated with development of severe HT (grade 3 or 4) during therapy, and the number of Xofigo treatments.

**Results:** Among 54 patients 7 (13 %) demonstrated no pre-existing HT (grade 0). These patients developed only mild HT (grade 1: 56 %; grade 2: 22 %) and no severe HT. 37 patients (69 %) presented with initial grade 1 HT. This group showed increasing HT in 48 % (grade 2: 35 %, grade 3: 11 %, grade 4: 3 %). Grade 2 initial HT occurred in 7 patients (13 %). Here HT grades showed a tendency to grade 3 (total grade 3: 43 %, grade 2: 43 %) during therapy in all categories (Hb: 42.9 %; Plts: 42.9 %; Leuk: 28.6 %). Only one patient had an initial HT grade 3, regarding his Hb values. This patient did not show a change in HT level. Pre-existing HT correlated significantly with treatment-induced HT (R = 0,47), with significantly different HT levels between groups (P < 0,02) and significantly increased severe HT frequency (P < 0,005). Number of Xofigo treatment cycles was significantly different between groups: group 0: 6; group 1: 5,5; group 2: 4,7; group 3: 4 (P < 0,05).

**Conclusion:** Higher grades of HT before Xofigo therapy were significantly related to the development of severe HT during therapy, mainly affecting hemoglobin values, thus leading to increasing numbers of premature termination of Xofigo treatment.

**References**

1. Prostate cancer: ESMO Clinical Practice Guidelines for diagnosis, treatment and follow-up : Ann Oncol first published online June 27, 2013 doi:10.1093/annonc/mdt208.

## A8 QDOSE – comprehensive software solution for internal dose assessment

### Wencke Lehnert, Karl Schmidt, Sharok Kimiaei, Marcus Bronzel, Andreas Kluge

#### ABX-CRO advanced pharmaceutical services (Forschungsgesellschaft mbH), Dresden, Germany

**Aim:** The development of our QDOSE software was inspired by the need for a versatile, easy-to-use dosimetry software solution. Our experience in numerous clinical trials drove the design of QDOSE to incorporate all functionality within one stand-alone system. It has a fully integrated workflow, validated methodology and advanced output functionality.

**Methods:** QDOSE combines all dosimetry steps into a single software. This includes loading of imaging data, 2D and 3D coregistration, region and volume of interest (ROI / VOI) drawing and segmentation, curve fitting for time activity curves (TACs), calculation of cumulated activities and dose calculation.

Dose calculation entails (1) export of residence times to the OLINDA/EXM software [1], (2) spherical model dose calculation [2] implemented as continuous interpolation model and (3) Voxel S dose calculation. It will also include MIRD / ICRP phantom-based safety dosimetry calculation.

QDOSE incorporates all major workflows: (1) Planar Dosimetry featuring quantitative methodology according to [3]; (2) Hybrid Dosimetry using multiple time points of conjugate view imaging and one quantitative SPECT/CT imaging time point for improved quantification; (3) 3D Dosimetry using multiple time points of quantitative SPECT/CT or PET/CT imaging; and (4) Selective Internal Radiation Therapy (SIRT) Dosimetry providing post-treatment dosimetry using a single time point of 90Y PET/CT or Bremsstrahlung SPECT/CT as well as treatment planning using a 99mTc-MAA SPECT/CT scan.

QDOSE also features direct comparison of dose estimates for the different available workflows as well as dose prediction for treatment planning and nuclide comparison.

**Results:** QDOSE is validated extensively for use in our clinical trials. Technical validation was performed using synthetically generated digital phantom data for testing all major functionalities and calculations. Further workflow validation was performed using a number of patient datasets. Dose calculations for the spherical model and Voxel S were validated against the spherical model implemented in the OLINDA/EXM software [1, 2] using digital phantoms containing spherical objects. Clinical validation using a variety of clinical data is done in collaboration with a number of leading internal radionuclide therapy sites.

**Conclusion:** QDOSE is a versatile stand-alone, user-friendly, vendor-neutral dosimetry tool. It offers flexibility allowing for patient-specific dosimetry in a variety of clinical settings complemented with a streamlined workflow guidance saving the user time and reducing user errors.

**References**

[1] Stabin, M. G., Sparks, R.B., Crowe, E. OLINDA/EXM: the second-generation personal computer software for internal dose assessment in nuclear medicine. J Nucl Med, 46(6):1023–1027, 2005.

[2] Stabin, M. G., Konijnenberg, M. Re-evaluation of Absorbed Fractions for Photons and Electrons in Small Spheres. J Nucl Med, 41: 149–160, 2000.

[3] Siegel, J. A. et al. MIRD pamphlet no. 16: Techniques for quantitative radiopharmaceutical biodistribution data acquisition and analysis for use in human radiation dose estimates. J Nucl Med, 40(2):37S-61S, 1999.

## A9 Clinical impact of Time-of-Flight on next-generation digital PET imaging of Yttrium-90 radioactivity following liver radioembolization

### CL Wright^1^, K Binzel^1^, J Zhang^1^, Evan Wuthrick^2^, Piotr Maniawski^3^, MV Knopp^1^

#### ^1^Wright Center of Innovation in Biomedical Imaging, The Ohio State University, Columbus, OH, USA; ^2^Radiation Oncology, Wexner Medical Center at The Ohio State University, Columbus, OH, USA; ^3^Clinical Science - Nuclear Medicine, Philips Healthcare, Cleveland, OH, USA

**Aim:** The selection and utilization of Yttrium-90 (90Y) microspheres as a targeted radiotherapeutic is driven by the existing clinical need to selectively embolize tumor-induced vasculature and deliver high doses of therapeutic radioactivity to unresectable liver lesions. This is particularly important when external beam radiation therapy is deemed impractical. Bremsstrahlung imaging currently serves as the standard for post-90Y radioembolization assessment but this approach is limited with poor image quality, poor 90Y-to-background contrast, and limited capability for post-therapy quantitative dosimetry. Although it emits high-energy electrons for therapeutic purposes, 90Y is a theranostic agent given that it also produces a small fraction of discrete annihilation photons which are imagable with conventional photomultiplier tube-based PET technology (cPET/CT, [1]). A recent technological innovation has replaced these conventional photomultiplier tubes in the PET gantry with next-generation, solid-state, digital photon counting PET detectors (digital PET or dPET) which have Time-of-Flight capability as well as markedly improved timing resolution. Our aim is to assess the clinical impact of Time-of-Flight on 90Y dPET/CT image quality and post-therapy assessment in patients following liver radioembolization.

**Methods:** In an ongoing trial, we are using a pre-commercial release digital PET/CT system (Vereos, Philips Healthcare) to image and assess 90Y microsphere biodistribution in patients with malignant/metastatic liver lesions after radioembolization therapy. Bremsstrahlung SPECT/CT imaging was performed within a few hours immediately following radioembolization with a total image acquisition time of 22 m. Digital PET imaging was performed in 10 patients at 4 – 50 h following liver radioembolization with a total image acquisition time of 21 m. PET data were reconstructed using a 3D OSEM algorithm with and without Time-of-Flight using a voxel size of 4x4x4 mm3. Image characteristics and isocontour volumes of 90Y activity were then assessed by matched comparison using the Intellispace Portal workstation (Philips). In addition, matched comparison with standard bremsstrahlung SPECT/CT imaging (Symbia T16, Siemens Healthcare) was performed to further assess the practicality and clinical utility of this dPET/CT imaging approach.

**Results:** For all patients, dPET images were rated as evaluable and, when compared with bremsstrahlung SPECT/CT, dPET detection of 90Y radioactivity enabled markedly improved qualitative and volumetric assessment of 90Y microsphere biodistribution. In particular, dPET/CT technology enables post-therapy 90Y imaging with shorter image acquisition times, improved image quality, contrast, and volumetric assessment when compared with standard bSPECT/CT approaches. Furthermore, Time-of-Flight improved overall image quality and 90Y-to-background contrast for dPET/CT images when compared with non-Time-of-Flight dPET but there were no significant differences in the measured intrahepatic volumes of 90Y activity using 1 % isocontours.

**Conclusion:** There remains an unmet clinical need to improve qualitative and quantitative imaging assessment of 90Y-based radiotherapies. Current imaging approaches for assessing 90Y in vivo are (1) challenging in terms of low quality / poorly quantitative bremsstrahlung techniques and (2) potentially disruptive to clinical workflows given the long image acquisition times needed for current cPET techniques. We demonstrate that Time-of-Flight dPET image quality is superior to the non-Time-of-Flight dPET and standard bremsstrahlung approaches. Using dPET technology in the future, we expect that new strategies for 90Y image acquisition as well as new reconstruction methodologies will facilitate even shorter image acquisition times with better image quality and more accurate dose quantification in patients treated with existing and new 90Y-based theranostics.

**References**

1. Wright, C.L., et al., Theranostic Imaging of Yttrium-90. Biomed Res Int, 2015. 2015: p. 481279.

## A10 Snakes in patients! Lessons learned from programming active contours for automated organ segmentation

### M Blaickner^1^, E Rados^1^, A Huber^1^, M Dulovits^2^, H Kulkarni^3^, S Wiessalla^3^, C Schuchardt^3^, RP Baum^3^, B Knäusl^4^, D Georg^4^

#### ^1^AIT Austrian Institute of Technology, Health & Environment Department -Biomedical Systems, Vienna Austria; ^2^Woogieworks Animation Studio, Perchtoldsdorf, Austria; ^3^THERANOSTICS Center for Molecular Radiotherapy and Molecular Imaging (PET/CT) ENETS Center of Excellence, Zentralklinik Bad Berka, Germany; ^4^Department of Radiation Oncology, Division of Medical Radiation Physics, Christian Doppler Laboratory for Medical Radiation Research for Radiation Oncology, Medical University of Vienna, Vienna, Austria

**Aim:** Individualized dosimetry calculations in external beam- or radionuclide therapy require individual organ masses. Organ segmentation is either done by manual delineation or (semi)-automated algorithms. The former is dreadfully time consuming and prone to interobserver errors whereas the later still lacks a golden standard, especially in case of low image contrast. This talk gives an overview of the work with active contours (snakes!) as a method for automated organ segmentation.

**Methods:** Several versions of active contours were implemented and tested, e.g. two-dimensional (2D) snakes with a closed curve and a three-dimensional (3D) where the active contour is represented as a subdivision surface (SubD). The external force is computed as gradient vector flow (GVF), i.e. the diffusion of the gradient vectors. In-house snakes were implemented in Python and SideFX Houdini as well as existing active contours used, such as the corresponding function in Adobe After Effects. The rigidity of the active contour was varied as well as a node network in Houdini implemented, containing multiple image preprocessing modules. The different snakes were evaluated on single- and multi- modality patient imaging and compared to manual contouring.

**Results:** In-house implemented 2D snakes have shown good performance even on low contrast images such as soft tissue organs on CT without contrast media. However the slice by slice approach faces difficulties at the top and bottom of organs. This can be improved by means of 3D SubDs as active contours, though only for specific image parameters, e.g. Ga68-DOTA PET images of the liver. For CT data of liver and kidney without contrast media the volume comparison between manual and 2D segmentation yielded for the kidney a mean deviation of 5.16 % and for the liver 3.09 %. The dice coefficient was on average 0.89 and 0.94 respectively. Regarding 3D segmentation the deviation of the kidney volumes was on average 18.68 % and for the liver 13.02 %, where the dice coefficient was 0.69 and 0.83. Poor resolution of the CT images in z-direction considerably worsens the performance of the 3D active contour. Distracting forces due to inhomogeneous grey values within the organs and low contrast to neighbouring structures requires extensive image pre-processing. Computing the snake’s rigidity has a high impact on the performance with concave regions.

**Conclusion:** Even with a versatile tool like active contours and GVF automated segmentation of soft tissue organs in low contrast remains challenging. A best practice model requires the combination of elaborate pre-processing, 2D as well as 3D active contours.

**References**

1) R. J. H. M. Steenbakkers, J. C. Duppen, I. Fitton, K. E. I. Deurloo, L. J. Zijp, E. F. I. Comans, A. L. J. Uitterhoeve, P. T. R. Rodrigus, G. W. P. Kramer, J. Bussink, K. De Jaeger, S. a Belderbos, P. J. C. M. Nowak, M. van Herk, and C. R. N. Rasch, “Reduction of observer variation using matched CT-PET for lung cancer delineation: a three-dimensional analysis,” Int J Radiat Oncol Biol Phys, vol. vol 64, no 2, pp. pp. 435–48, Feb. 2006.

2) C. Xu and J. L. Prince, “Gradient vector flow: a new external force for snakes,” in, 1997 IEEE Computer Society Conference on Computer Vision and Pattern Recognition, 1997. Proceedings, 1997, pp. 66–71.

## A11 Influence of a genetic polymorphism on brain uptake of the dual ABCB1/ABCG2 substrate [11C]tariquidar

### M Bauer^1^, B Wulkersdorfer^1^, W Wadsak^2^, C Philippe^2^, H Haslacher^3^, M Zeitlinger^1^, O Langer^1,4^

#### ^1^Department of Clinical Pharmacology, Medical University of Vienna, Vienna, Austria; ^2^Department of Biomedical Imaging and Image-guided Therapy, Medical University of Vienna, Vienna, Austria; ^3^Department of Laboratory Medicine, Medical University of Vienna, Vienna, Austria; ^4^Health and Environment Department, AIT Austrian Institute of Technology GmbH, Seibersdorf, Austria

**Aim:** Transport of drugs by membrane transporters is an important source of inter-individual variations in drug distribution, drug response and of major concern regarding drug-drug interactions. The investigational third-generation P-glycoprotein (ABCB1) inhibitor tariquidar has been used in clinical trials in tumour patients to overcome multidrug resistance. [11C]Tariquidar is transported at tracer doses by ABCB1 and breast cancer resistance protein (ABCG2) and can be used to measure the functional activities of these two transporters at the blood–brain barrier (BBB) [1]. Carriers of a loss-of-function single nucleotide polymorphism (SNP) in the ABCG2 gene (c.421C > A, Q141K) have altered plasma pharmacokinetics of ABCG2 substrate drugs as compared to subjects without the genetic variation [2]. We aim to assess the effect of this ABCG2 SNP on brain distribution of [11C] tariquidar.

**Methods:** Healthy male volunteers were genotyped for the ABCG2 SNP and allocated to a c.421C/C (wild-type) and c.421C/A (heterozygous) group, respectively (n = 5 per group). Subjects underwent two consecutive 60-min dynamic brain PET scans with [11C]tariquidar, a first scan after administration of a tracer dose of [11C]tariquidar (<20 μg) and a second scan during continuous i.v. infusion of unlabelled tariquidar (3.75 mg/min) in order to inhibit ABCB1 at the BBB. Atlas based region of interest whole brain grey matter was defined on individual magnetic resonance images and time-activity curves extracted from the co-registered dynamic PET images. Arterial blood samples were collected and radioactivity in plasma was measured in a gamma counter. The ratio of the area under the time-activity curves (AUC) in brain and plasma (AUCbrain/AUCplasma) was calculated for the first and second PET scan.

**Results:** In the first PET scan, no significant differences in AUCbrain/AUCplasma between the c.421C/C group (mean ± SD = 0.16 ± 0.05) and the c.421C/A group (0.15 ± 0.02) were observed (p = 0.944, Mann Whitney-U test). During ABCB1 inhibition (scan two) AUCbrain/AUCplasma was not significantly different compared to scan 1 for the c.421C/C group (AUCbrain/AUCplasma: 0.18 ± 0.07) but significantly increased for the c.421C/A group (AUCbrain/AUCplasma: 0.23 ± 0.04, +42 ± 8 % %, p = 0.008, Mann Whitney-U test).

**Conclusion:** Our data suggest that the effect of the ABCG2 SNP on brain distribution of the dual ABCB1/ABCG2 substrate [11C]tariquidar was masked in the first PET scan by the functional compensation between ABCB1 and ABCG2 at the BBB. Only after ABCB1 inhibition the ABCG2 SNP was found to have a significant effect on brain distribution of [11C]tariquidar. To our knowledge, this is the first study showing functional relevance of a polymorphism in the ABCG2 gene on brain distribution of an ABCG2 substrate. Our findings may have important implications for treatment of carriers of this SNP with ABCG2 substrate drugs. Moreover, our data highlight the suitability of our PET protocol to measure ABCG2 function at the human BBB.

**References**

1. Wanek T, Kuntner C, Bankstahl JP, et al. A novel PET protocol for visualization of breast cancer resistance protein function at the blood–brain barrier. J Cereb Blood Flow Metab. 2012;32:2002–2011.

2. Ieiri I. Functional Significance of Genetic Polymorphisms in P-glycoprotein (MBR1, ABCB1) and Breast Cancer Resistance Protein (BCRP, ABCG2). Drug Metab Pharmacokinet. 2012;27:85–105.

## A12 Outcome prediction of temporal lobe epilepsy surgery from P-glycoprotein activity. Pooled analysis of (R)-[11C]-verapamil PET data from two European centres

### M Bauer^1,2^, M Feldmann^2,3^, R Karch^4^, W Wadsak^5^, M Zeitlinger^1^, MJ Koepp^3^, M-C Asselin^2^, E Pataraia^6^, O Langer^1^

#### ^1^Department of Clinical Pharmacology, Medical University of Vienna, Vienna, Austria; ^2^Wolfson Molecular Imaging Centre, University of Manchester, Manchester, UK; ^3^University College London, London, UK; ^4^Center for Medical Statistics, Informatics, and Intelligent Systems, Medical University of Vienna, Vienna, Austria; ^5^Department of Biomedical Imaging and Image-guided Therapy, Medical University of Vienna, Vienna, Austria; ^6^Department of Neurology, Medical University of Vienna, Vienna, Austria

**Aim:** Overexpression of the ABC-transmembrane transporter P-glycoprotein (Pgp) at the blood–brain barrier contributes to therapy refractory temporal lobe (TL) epilepsy [1]. Surgical removal of TL structures may result in favorable seizure outcome in approximately 60 % of operated cases [2]. Findings from a pilot PET study investigating Pgp function suggest that Pgp overactivity in the TL prior to surgery may be indicative of optimal postoperative outcome [3].

**Methods:** Pharmacoresistant TL epilepsy patients underwent PET scanning before surgery with the Pgp substrate radiotracer (R)-[11C]verapamil (VPM) in Manchester (n = 6, median age 44, range 18–56 years) and Vienna (n =8, range 33–54 years, dataset from previous study [3] extended). PET data were acquired on the Siemens High Resolution Research Tomograph in Manchester and on the GE Advance PET scanner in Vienna. Atlas based regions of interest were defined in the ipsilateral TL on individual magnetic resonance images and time-activity curves extracted from the co-registered dynamic PET images [1,3]. Metabolite corrected arterial input functions were used to estimate the net influx of VPM from plasma into brain (K1) from the first 10 min of PET data [1,3]. Clinical follow-up data on seizure activity and anti-epileptic medication were available for 4 ± 1 years for Manchester and 6 ± 1 years for Vienna patients. Ranking of surgery outcome was done according to the modified Wieser’s classification (best outcome assigned rank 1) [3].

**Results:** Correlation of VPM K1 values in ipsilateral TL with surgery outcome rank in individual patients was highly positive (Pearson r = 0.7573, p = 0.0017, n = 14). Patients with the highest Pgp activity (lowest K1 values) before surgery showed optimal surgery outcome (seizure and medication free) whereas those with the lowest Pgp activity continued to have seizures and to take medication even after surgery. Findings were consistent for both centres.

**Conclusion:** Pooled analysis of pre-operative VPM PET data consistently predicted outcome after TL epilepsy surgery in two centres, confirming findings from a previous pilot study. PET measurement of Pgp function should be further evaluated as an imaging biomarker for the prediction of TL epilepsy surgery outcome.

**References**

[1] Feldmann M, Asselin M-C, Liu J, Wang S, McMahon A, Anton-Rodriguez J, Walker M, Symms M, Brown G, Hinz R, Matthews J, Bauer M, Langer O, Thom M, Jones T, Vollmar C, Duncan JS, Sisodiya SM, Koepp MJ. P-glycoprotein expression and function in patients with temporal lobe epilepsy: a case–control study. Lancet Neurol, 2013; 12: 777–785

[2] Téllez-Zenteno JF1, Dhar R, Wiebe S. Long-term seizure outcomes following epilepsy surgery: a systematic review and meta-analysis. Brain, 2005; 128:1188–1198

[3] Bauer M, Karch R, Zeitlinger M, Liu J, Koepp MJ, Asselin M-C, Sisodiya SM, Hainfellner JA, Wadsak W, Mitterhauser M, Müller M, Pataraia E, Langer O. In vivo P-glycoprotein function before and after epilepsy surgery. Neurology, 2014; 83:1326–1331

## A13 In-vitro and in-vivo characterization of [18F]FE@SNAP and derivatives for the visualization of the melanin concentrating hormone receptor 1

### M Zeilinger^1^, C Philippe^1^, M Dumanic^1^, F Pichler^1^, J Pilz^1^, M Hacker^1^, W Wadsak^1^, M Mitterhauser^1,2^

#### ^1^Department of Biomedical Imaging and Image-guided Therapy, Division of Nuclear Medicine, Radiopharmacy and Experimental Nuclear Medicine, Medical University of Vienna, Vienna, Austria; ^2^Ludwig Boltzmann Institute for Applied Diagnostics, Vienna, Austria

**Aim:** The melanin concentrating hormone (MCH) is a cyclic neuropeptide which is predominantly expressed in the lateral hypothalamus and zona incerta [1, 2]. The biological effect is mediated by two G-protein coupled receptors (GPCR), the MCH receptor 1 and 2 (MCHR1 and MCHR2) [3]. Changes in the expression of the MCHR1 have been shown to be involved in a variety of pathologies, such as depression and anxiety disorders and are related to the central mechanisms of obesity [4, 5]. In this regard, the preparation and first in-vitro studies of [18F]FE@SNAP and [11C]SNAP-7941, as potential positron emission tomography (PET) radiotracers for the visualization of the MCHR1, were performed successfully [6, 7, 8, 9]. Based on the preceding results the present present study aimed at a further in-vitro and in-vivo evaluation of [18F]FE@SNAP and its structurally closed non radioactive derivatives FE@SNAP, SNAP-7941 and (+)-SNAP-7941 to provide high-impact predictive values for a further translation into clinical PET imaging.

**Methods:** In-vitro: Binding affinity of SNAP-7941, (S)-SNAP-7941, FE@SNAP and MCH were determined in competitive centrifugation binding studies with [125I]MCH using chinese hamster ovary (CHO-K1) membranes stably expressing the human MCHR1. Kinetic real-time binding studies with [18F]FE@SNAP were conducted on adherent CHO-K1 cells stably expressing the human MCHR1 and MCHR2. Experiments on blank CHO-K1 cells were performed to determine unspecific binding.

In-vivo: Small-animal μPET/CT and μMRI imaging was performed in naïve male Sprague Dawley rats (HIM:OFA; 438 ± 42 g). The rats were anesthetized with isoflurane/oxygen (1.5 % / 0.8 mL) and [18F]FE@SNAP was administered through the lateral tail vein (47.8 ± 1 MBq; SA: 19.7 ± 6GBq/μmol). Radiotracer uptake in the brain (SUV; g/mL) was determined under i) baseline conditions, ii) blocking with 15 mg/kg SNAP-7941 and iii) blocking with 15 mg/kg tariquidar. After the imaging the rats were sacrificed, the organs were harvested and subjected to radioactivity measurements. The resulting values were expressed as percent injected dose per gram tissue (%ID/g).

**Results:** Binding studies revealed Ki values of 6.25 ± 2.26nM (SNAP-7941; n = 3), 7.34 ± 0.57nM ((+)-SNAP-7941; n = 3), 20.74 ± 3.62nM (FE@SNAP; n = 3) and 0.16 ± 0.06nM (MCH; n = 3) respectively. Kinetic binding studies of [18F]FE@SNAP showed observed association rate constants of 0.069 ± 0.003 M-1 · min-1 (n = 3) to hMCHR1 and 0.063 ± 0.004 M-1 · min-1(n = 3) to hMCHR2. Kinetic binding studies with blank CHO-K1 cells revealed no radiotracer uptake & low unspecific binding (n = 3). In-vivo small animal experiments evinced a brain uptake of 0.28 ± 0.05 g/mL (baseline group; n = 5), 0.28 ± 0.03 g/mL (blocking with SNAP-7941; n = 2) and 0.48 ± 0.17 g/mL (blocking with tariquidar, n = 2).

**Conclusion:** The binding affinity of SNAP-7941 and the (S)-enantiomer revealed comparable results, both in a low nanomolar range. As opposed to this, FE@SNAP evinced a significantly lower Ki. Real-time binding studies of [18F]FE@SNAP showed no differences in the binding kinetics between the receptor subtypes. Specific uptake of [18F]FE@SNAP in the rat brain seems to be prevented by p-gp and/or BCRP, since small animal μPET experiments revealed a 2–2.5 fold increased brain uptake after p-gp/BCRP inhibition with tariquidar. Further in-vivo investigations with respect to peripheral MCHR1 expression of healthy, obese and diabetic rats need to be performed to further proof the applicability of the MCHR1 concept.

**References**

[1] Kawauchi, et al. (1983) Nature. 305:321–3.

[2] Bittencourt et al. (1992) J Comp Neurol. 319(2):218–45.

[3] Sailer et al. (2001) Proc Nat Acad Sci USA. 98:7564–7569.

[4] Smith et al. (2006) Neuropsychopharmacology. 31(6):1135–45.

[5] Ito et al. (2009) Eur J Pharmacol. 624(1–3):77–83.

[6] Philippe et al. (2012) Bioorg Med Chem. 20(19):5936–40.

[7] Philippe et al. (2012 Appl Radiat Isot. 70(10):2287–94.

[8] Philippe et al. (2013) Sci Pharm. 81(3):625–39.

[9] Philippe et al. (2013) Nuc Med Biol. 40(7):919–25.

## A14 Reducing time in quality control leads to higher specific radioactivity of short-lived radiotracers

### L Nics^1^, B Steiner^1^, M Hacker^1^, M Mitterhauser^2^, W Wadsak^1^

#### ^1^Department of Biomedical Imaging and Image guided Therapy, Division of Nuclear Medicine, Medical University of Vienna, Vienna, Austria; ^2^Ludwig Boltzmann Institute for Applied Diagnostics, Vienna, Austria

**Aim:** Time plays a pivotal role in the preparation of radiopharmaceuticals (RP) labeled with short-lived radionuclides (e.g. carbon-11, nitrogen-13, gallium-68). In particular, it has to be taken into account that radioactive decay starts to reduce available activity as soon as the produced radionuclide is delivered to the synthesis unit. This is also true for the specific radioactivity (SA; activity per mass unit) which is of significant importance for radiotracers targeting saturable targets such as transporters or receptors. This process lasts until application of the RP after passing the quality control (QC) and final release of the product. So far, time reduction was mostly implemented during the chemical synthesis process or subsequent purification. In contrast, QC procedures were not considered to a full extent in that perspective. Determination of radiochemical purity and SA normally requires the use of high performance liquid chromatography (HPLC) which is also the most time-consuming step in the QC procedure. Recently, Nakao et al. [1] suggested a method involving hybrid (ultra-)HPLC ((U)HPLC) to shorten run times. Hence, aim of this work was to implement fast HPLC for [11C]DASB and (+)-[11C]PHNO to enhance specific radioactivity.

**Methods:** [11C]DASB and (+)-[11C]PHNO were prepared according to methods described in detail elsewhere. [2,3] All analytical HPLC analyses were performed on an Agilent 1260 system equipped with a quaternary pump, a multi wavelength UV-detector, a manual injector and a radiodetector and controlling software. HPLC column was a Waters X-bridge BEH Shield RP-18 (4.6 x 50 mm, 2.5 μm, 130 Å). Injection volume was 5 μL. For [11C]DASB, the following conditions were applied: wavelength 254 nm; Flow rate 1.0 mL/min; Mobile phase: A: 50 mM ammonia phosphate buffer, pH 9.3; B: 90 % ACN / 10 % aqua dest. (v/v); Isocratic: 40 % A / 60 % B. For (+)-[11C]PHNO, the following conditions were applied: wavelength 280 nm; Flow rate 1.6 mL/min (0-30”), then 1.0 mL/min; Mobile phase: A: 100 mM ammonia phosphate buffer, pH 2.1 including 5 mM sodium-1-octasulfonate; B: 90 % ACN / 10 % aqua dest. (v/v); C: Aqua purificata (HPLC grade); D: 50 mM ammonia phosphate buffer, pH 9.3; Isocratic: 33 % A / 20 % B / 14 % C / 33 % D.

**Results:** For [11C]DASB, total HPLC run time was 1.0 min; precursor MASB eluted after 23 sec, DASB eluted after 55 sec, respectively. For (+)-[11C]PHNO, total HPLC run time was 1.1 min; precursor (+)-HNO eluted after 27 sec, PHNO eluted after 61 sec, respectively. Hence, a reduction of >5 min was achieved for both radiotracers which equals an increase in available activity and SA of more than 15 %.

**Conclusion:** This work demonstrates the importance of optimizing QC procedures such as HPLC to reduce overall time before release and application of short-lived radiopharmaceuticals. An increase in SA of >15 % was observed when implanting an optimized chromatographic protocol for [11C]DASB and (+)-[11C]PHNO.

**References**

[1] Nakao R et al. J Pharm Biomed Anal, 2009.

[2] Haeusler D et al. Appl Rad Isot, 2009.

[3] Rami-Mark C et al. Appl Rad Isot, 2013.

## A15 In vitro 11C-erlotinib binding experiments in cancer cell lines with epidermal growth factor receptor mutations

### A Traxl^1^, Thomas Wanek^1^, Kushtrim Kryeziu^2^, Severin Mairinger^1^, Johann Stanek^1,3^, Walter Berger^2^, Claudia Kuntner^1^, Oliver Langer^1,3^

#### ^1^Biomedical Systems, Health & Environment Department, AIT Austrian Institute of Technology GmbH, Seibersdorf, Austria; ^2^Institute of Cancer Research, Department of Medicine I, Medical University of Vienna, Vienna, Austria; ^3^Department of Clinical Pharmacology, Medical University of Vienna, Vienna, Austria

**Aim:** Erlotinib is a tyrosine kinase inhibitor of the epidermal growth factor receptor (EGFR) used to treat patients with non-small cell lung cancer (NSCLC). NSCLC patients with activating mutations (e.g. exon 19 deletion) in the EGFR gene have increased response rates to erlotinib treatment compared to patients with wild-type EGFR. However, patients with activating EGFR mutations eventually develop resistance to erlotinib caused by secondary mutations (e.g. T790M). 11C-erlotinib PET signal was found to be higher in NSCLC tumors with an activating EGFR mutation than in tumors with wild-type EGFR [1]. It is however, not known if 11C-erlotinib PET will be able to distinguish tumors with secondary resistance causing mutations from erlotinib-sensitive tumors. Aim of this study was to measure in vitro 11C-erlotinib binding in erlotinib-sensitive and erlotinib-resistance cancer cell lines.

**Methods:** One glioblastoma (U-87 MG) and four NSCLC (erlotinib-sensitive HCC827, erlotinib-resistant HCC827, gefitinib-resistant HCC827, and erlotinib-resistant HCC827 with a secondary EGFR mutation) cell lines were incubated with 11C-erlotinib up to 60 min. In a second set of experiments cell lines were pretreated with unlabeled erlotinib or different P-glycoprotein (P-gp) and breast cancer resistance protein (BCRP) inhibitors (elacridar, Ko143, tariquidar) and then incubated with 11C-erlotinib for 30 min. Retained radioactivity in the cells was measured in a gamma counter and normalized to cell count.

**Results:** Uptake of 11C-erlotinib was significantly lower in U-87 MG cells compared to all NSCLC cell lines. No significant differences were observed in the uptake of 11C-erlotinib between the four NSCLC cell lines. In all NSCLC cell lines 11C-erlotinib uptake was significantly reduced following pretreatment with unlabeled erlotinib, which was not the case for U-87 MG cells. Pretreatment with elacridar, Ko143 or tariquidar had no effect on 11C-erlotinib uptake in all tested cell lines.

**Conclusion:** We found significantly lower uptake of 11C-erlotinib in U-87 MG cells with wild-type EGFR compared to all NSCLC cell lines, which had activating EGFR mutations. No significant differences in 11C-erlotinib uptake were observed between erlotinib-sensitive, erlotinib-resistant, and gefitinib-resistant NSCLC cell lines. In all NSCLC cell lines a significant reduction of 11C-erlotinib uptake after pretreatment with unlabeled erlotinib was found, suggesting specific binding to EGFR. Efflux of 11C-erlotinib by P-gp and BCRP was not evident in any of the studied cell lines. Taken together, our findings suggest that 11C-erlotinib PET may be suitable to distinguish wild-type EGFR from EGFR with activating mutations, but unsuitable to identify secondary resistance causing mutations. To further extend our in vitro findings, PET studies will be carried out in tumor-xenografted nude mice using erlotinib-resistant and erlotinib-sensitive NSCLC cell lines.

**References**

[1] Bahce et al. Clin Cancer Res.2013 Jan 1; 19(1):183–93

## A16 7-[11C]methyl-6-bromopurine, a PET tracer to measure brain Mrp1 function: radiosynthesis and first PET evaluation in mice

### S Mairinger^1^, T Wanek^1^, A Traxl^1^, M Krohn^2^, J Stanek^1^, T Filip^1^, M Sauberer^1^, C Kuntner^1^, J Pahnke^2^, O Langer^1,3^

#### ^1^Health & Environment Department, AIT Austrian Institute of Technology GmbH, Seibersdorf, Austria; ^2^Department of Neuro-/Pathology, University of Oslo (UiO) and Oslo University Hospital (OUS), Oslo, Norway; ^3^Department of Clinical Pharmacology, Medical University of Vienna, Vienna, Austria

**Aim:** Multidrug resistance-associated protein 1 (MRP1) is a membrane transporter expressed at the blood–brain and blood-cerebrospinal fluid barriers, which has been suggested to play a role in beta-amyloid clearance from brain into blood [1]. 7-[11C]methyl-6-bromopurine ([11C]7m6BP) is a novel PET tracer to measure MRP1 function in the brain [2]. Visualization of MRP1 function with [11C]7m6BP is based on a pro-drug approach: the probe enters the brain by passive diffusion and gets rapidly converted into its glutathione conjugate S-(6-(7-methylpurinyl))glutathione ([11C]PSG), which is effluxed from the brain by MRP1. Aim of this work was to establish the radiosynthesis of [11C]7m6BP in our lab and perform a first PET evaluation of [11C]7m6BP in wild-type and Mrp1(−/−) mice.

**Methods:** [11C]7m6BP was produced by N-methylation of commercially available 6-bromopurine using [11C]methyl triflate in a Tracerlab FX C Pro synthesis module with slight modifications compared to the literature [2]. Female C57BL6 wild-type and Mrp1(−/−) mice aged 153–211 days (n = 6 per group) underwent 90 min dynamic [11C]7m6BP PET scans. Whole-brain time-activity curves were obtained and the efflux rate constant (keff) of radioactivity from brain was calculated using data from 17.5-80 min after radiotracer injection. In separate groups of wild-type and Mrp1(−/−) mice radiolabelled metabolites of [11C]7m6BP were analyzed by radio-thin layer chromatography (TLC) in plasma and brain at 15 and 60 min after radiotracer injection (n = 3 per group and time point).

**Results:** [11C]7m6BP was obtained in a radiochemical yield of 16.8 ± 1.7 % (n = 12) (based on [11C]methyl triflate, decay-corrected) in a total synthesis time of 29 min. Radiochemical purity was >98 % and specific activity at end of synthesis was 428 ± 232 GBq/μmol (n = 8). Radio-TLC analysis showed that at 15 min after radiotracer injection all radioactivity in the brain consisted of the desired glutathione conjugate [11C]PSG. Whole brain time-activity curves were characterized by a rapid washout of radioactivity in wild-type mice and a prolonged retention of radioactivity in Mrp1(−/−) mice. Brain efflux rate constant (keff) of radioactivity was significantly (p < 0.05, t-test) reduced in Mrp1(−/−) compared to wild-type mice ((keff (h-1): wild-type: 1.29 ± 0.33; Mrp1(−/−): 0.25 ± 0.03).

**Conclusion:** We were able to set up the automated radiosynthesis of [11C]7m6BP in practical radiochemical yield and high radiochemical purity. A first PET evaluation suggests that [11C]7m6BP is suitable to measure Mrp1 function in mouse brain.

**Funding:** FWF-DACH project I 1609-B24.

**References**

[1] Krohn M. et al., J Clin Invest 2011, 121: 3924–31

[2] Okamura T. et al., Journal of Cerebral Blood Flow & Metabolism 2009, 29:504–11

## A17 18F labeled azidoglucose derivatives as “click” agents for pretargeted PET imaging

### D Svatunek^1^, C Denk^1^, M Wilkovitsch^1^, T Wanek^2^, T Filip^2^, C Kuntner-Hannes^2^, J Fröhlich^2^, H Mikula^1^

#### ^1^Institute of Applied Synthetic Chemistry, TU Wien, Vienna, Austria; ^2^Austrian Institute of Technology, Vienna, Austria

**Aim:** Bioorthogonal ligations are capable of forming covalent bonds in highly complex environments. Since their introduction in 2000 their potential to be used in pretargeting experiments has drawn much attention in the field of (PET-) imaging. (1) Pretargeting means splitting of the tracing compound into a marker, which is enriched in target tissue and a radiolabeled pull down reagent (PDR). By using such an approach, longer periods of time for enrichment of the marker compound can be provided, which is crucial for PET measurements with high contrast of target to normal tissue. After this labeling step the PDR is injected, which should react with the trapped marker in a bioorthogonal way. This would allow the use of short living radioisotopes in PET imaging of slow accumulating markers and leads to enhanced signal-to-noise ratios and lower radioactivity doses.

The most prominent bioorthogonal reaction, the strain-promoted azide alkyne cycloaddition (SPAAC), has been used in a wide variety of applications in chemical biology and biomedicine. (2) This reaction features the Huisgen-Cycloaddition between an azide and a strained cycloalkyne to form triazoles. SPAAC has already been used in pretargeted PET imaging featuring short lived 18F as radionuclide by Lee et al. (3) although they do not report about biodistribution and stability of the azide-labeled pull-down-reagent.

Aim of this work was the synthesis and in vivo evaluation of 18F labeled azidoglucoses as pull-down reagent for pretargeted PET imaging using SPAAC as bioorthogonal ligation. Using glucose derivatives as PDRs has several advantages, such as good and fast biodistribution and possible active uptake into cells by glucose transporters. Blocking of the 6 position with 18F will prevent phosphorylation and therefore trapping in cells can only occur after bioorthogonal reaction. Two different isomers were evaluated, both containing 18F at position 6 while either 1-OH (18F-AzFDG16) or 2-OH (18F-AzFDG26) was displaced against an azide group.

**Methods:** Precursors for radiosynthesis were prepared in 4 and 3 steps respectively starting from commercially available material. Radiosynthesis were carried out by nucleophilic displacement of a nosylate by 18F-Fluorine followed by alkaline hydrolysis on a tC18 SepPak cartridgeand formulation in saline solution.

In vivo studies were performed by administration of 18F labeled azidoglucoses to female BALB/c mice followed by dynamic PET imaging (n = 6 for 18F-AzFDG16, n = 4 for 18F-AzFDG26) for 120 min. At the end of the imaging experiment the mice were sacrificed and harvested urine and plasma samples were screened for radiometabolites using radio-thin-layer-chromatography (TLC). Selected organs were withdrawn and gamma counted to validate data from the last PET time frame.

**Results:** 18F-AzFDG16 and 18F-AzFDG26 were obtained in good yields and high radiochemical purity. PET Imaging revealed a very homogenous and fast biodistribution of both substances in all regions in the body excluding the brain, as well as rapid renal excretion. Furthermore, PET imaging revealed no significant extent of in vivo defluorination of both PDRs, since no enhanced radioactivity uptake in bone was observed. High Stability of these reagents was also confirmed by metabolite analysis (radio-TLC).

**Conclusion:** Fluoroazidoglucoses have proven to be promising click agents for in vivo application applying SPAAC, due to homogenous and fast biodistribution, high metabolic stability as well as fast excretion. Synthesis and evaluation of further derivatives as well as in vivo click experiments are currently under investigation.

**References**

(1) Knight, J. C.; Cornelissen, B. Am. J. Nucl. Med. Mol. Imaging 2014, 4 (2), 96–113.

(2) Baskin, J. M.; Prescher, J. A.; Laughlin, S. T.; Agard, N. J.; Chang, P. V; Miller, I. A.; Lo, A.; Codelli, J. A.; Bertozzi, C. R. Proc. Natl. Acad. Sci. U. S. A. 2007, 104, 16793–16797.

(3) Lee, S. B.; Kim, H. L.; Jeong, H.-J.; Lim, S. T.; Sohn, M.-H.; Kim, D. W. Angew. Chem. Int. Ed. Engl. 2013, 52 (40), 10549–10552.

## A18 Bioorthogonal tools for PET imaging: development of radiolabeled 1,2,4,5-Tetrazines

### C Denk^1^, D Svatunek^1^, T Wanek^2^, S Mairinger^2^, J Stanek^2^, T Filip^2^, J Fröhlich^1^, H Mikula^1^ and C Kuntner-Hannes^2^

#### ^1^Institute of Applied Synthetic Chemistry, TU Wien, Vienna, Austria; ^2^Austrian Institute of Technology, Biomedical Systems, Vienna, Austria

**Aim:** Slow tracer accumulation of imaging probes can be circumvented by pretargeting using bioorthogonal ligations. These fast and biocompatible “click” reactions are capable of forming a covalent linkage between a pre-administered marker and a labeled pull down reagent (PDR) in vivo. Due to particular high ligation rates the inverse electron demand Diels-Alder reaction (IEDDA) of strained dienophiles with 1,2,4,5-tetrazines (Tz) has gained interest in nuclear medicine.

Until recently, only few radiolabeled tetrazines mainly based on radiometals and bulky chelating agents have been reported. However, the use of short-lived PET isotopes (18F, 11C) with favorable decay characteristics and high specific activity was limited due to chemical stability and reactivity restrictions.

Within this contribution an overview will be given describing the concept of bioorthogonal PET imaging. Furthermore the development of various low molecular weight radiotetrazine probes labeled with fluorine-18 and carbon-11 will be presented.

**Methods:** In silico methods were used to investigate IEDDA ligation reactivity prior to synthesis. Tetrazines were synthesized using metal catalysis, and precursor substances (tosylates for 18F-radiochemistry, primary amines for 11C-radiochemistry) were labeled in good radiochemical yields avoiding the use of prosthetic groups. Radiochemical purity was assessed by radio-HPLC and TLC, and the identity of labeled PDRs was verified by co-injecton with non-radioactive reference materials. Biodistribution and excretion kinetics were investigated by administration into female BALB/c mice and whole body dynamic PET/MR scanning for 120 min (18F) or 60 min (11C). Furthermore blood and urine samples were analyzed for radiometabolites and gamma counting of selected organs was conducted to confirm PET results.

**Results:** A set of several tetrazine probes labeled with fluorine-18 and carbon-11 was developed. The compounds 3-(3-[18F]-Fluoropropyl)-6-methyl-1,2,4,5-tetrazine and N-[11C]-methyl-1-(6-methyl-1,2,4,5-tetrazin-3-yl)methanamine exhibit high metabolic stability, homogenous biodistribution (including the brain) and fast excretion making them valuable candidates to be used as PDR in pretargeted PET applications. Other 18F-tetrazines were found to be metabolically unstable but still proved highly valuable to be used for rapid radiolabeling of dienophile-tagged biomolecules.

**Conclusion:** To this end a selection of promising bioorthogonal tools is available that should allow for pretargeted PET imaging of slow accumulating probes as well as rapid radiolabeling in highly complex (biological) matrices.

**References**

1. D. M. Patterson, L. A. Nazarova, J. A. Prescher, ACS Chemical Biology 2014.

2. Z. Li, H. Cai, M. Hassink, M. L. Blackman, R. C. D. Brown, P. S. Conti, J. M. Fox, Chemical Communications 2010, 46, 8043–8045.

3. C. Denk, D. Svatunek, T. Filip, T. Wanek, D. Lumpi, J. Fröhlich, C. Kuntner, H. Mikula, Angewandte Chemie International Edition 2014, 53, 9655–9659.

## A19 Preclinical evaluation of [18F]FE@SUPPY- a new PET-tracer for oncology

### T Balber^1^, J Singer^1,2,3^, J Fazekas^2,3^, C Rami-Mark^1^, N Berroterán-Infante^1^, E Jensen-Jarolim^2,3^, W Wadsak^1^, M Hacker^1^, H Viernstein^4^, M Mitterhauser^1^

#### ^1^Biomedical Imaging and Image-Guided Therapy, Division of Nuclear Medicine, Medical University of Vienna, Vienna, Austria; ^2^Department of Immunology and Oncology, Institute of Pathophysiology and Allergy Research, Center of Pathophysiology, Infectiology and Immunology, Medical University of Vienna, Vienna, Austria; ^3^Department of Comparative Medicine, Messerli Research Institute of the University of Veterinary Medicine Vienna, Medical University Vienna and University Vienna, Vienna, Austria; ^4^Department of Pharmaceutical Technology and Biopharmaceutics, Faculty of Life Sciences, University of Vienna, Vienna, Austria

**Aim:** The human adenosine-3-receptor (hA3R) is highly expressed in various tumor cell lines. (1) [18F]FE@SUPPY, a hA3R antagonist with high affinity and selectivity, was developed as a potential PET-tracer for tumor imaging. The preclinical evaluation of this tracer aimed at the determination of the specific binding to tumor tissue compared to healthy tissue. Moreover, a further aim was the identification of a suitable human tumor cell line for transplantation into immunocompromised mice (xenograft model).

**Methods:** Characterization of tumor cell lines regarding hA3R protein expression was conducted using Western Blot, flow cytometry and immunofluorescent staining. Specific binding of [18F]FE@SUPPY was investigated in autoradiography experiments using colorectal carcinoma tissue and healthy mucosa tissue derived from the same patient.

**Results:** The hA3R protein was detected via Western Blot in different tumor cell lines (A431, BT474, HT29 and PC3). Flow cytometry experiments revealed mean fluorescence intensity values (ΔMFI) of 113.6 ± 52 for A431, 53.6 ± 22 for HT29, 46.13 ± 18For BT474 and 49.1 ± 31 for PC3 cells, respectively (n ≥ 3). [18F]FE@SUPPY showed increased binding to tumor tissue (9.18 ± 2.38 fmol/mm2) compared to healthy tissue (3.50 ± 0.95 fmol/mm2).

**Conclusion:** Autoradiography experiments revealed a 2.6 fold increased uptake of [18F]FE@SUPPY in colorectal carcinoma tissue compared to healthy mucosa. The tumor cell lines HT29 and A431 showed the highest expression of the hA3R and therefore will be used for the planned xenograft model.

**References**

(1) Madi L et al. 2004 Clin Cancer Res; 10:4472–9.

## A20 Investigation of Small [18F]-Fluoroalkylazides for Rapid Radiolabeling and In Vivo Click Chemistry

### C Denk^1^, D Svatunek^1^, B Sohr^1^, H Mikula^1^, J Fröhlich^1^, T Wanek^2^, C Kuntner-Hannes^2^, T Filip^2^

#### ^1^Institute of Applied Synthetic Chemistry, TU Wien, Vienna, Austria; ^2^Austrian Institute of Technology, Biomedical Systems, Vienna, Austria

**Aim:** In recent years the concept of pretargeted PET imaging using bioorthogonal chemistry has drawn a lot of attention as it allows imaging of slow accumulating probes with short-lived radionuclides leading to enhanced signal-to-noise ratios and lower patient radiation doses. The underlying idea is based on a two-step approach that includes administration of a target tissue (e.g. tumor) sensitive probe followed by a radiolabeled pull down reagent (PDR). The probe and PDR will ligate in vivo via a bioorthogonal reaction, which does not interfere with native biochemical processes and exhibits high reaction rates.

The aim of this work was to investigate the biostability and -distribution of various low molecular-weight [18F]-fluoroalkylazides to evaluate their potential as PDRs in the bioorthogonal copper-free-click reaction (CFC) between azides and cyclooctynes.

**Methods:** Precursor materials for [18F]-fluoroalkylazides were obtained via straightforward synthetic chemistry using commercially available reagents. Radiolabeling of an improved precursor (crystalline instead of liquid) was achieved in high yields and short reaction times using the Kryptofix 222/K2CO3 method. The identity of target compounds was verified by comparison of HPLC retention times with non-radioactive reference materials.

In vivo biodistribution studies were performed by administration of [18F]-radiolabeled fluoroalkylazides to female BALB/c mice and subsequent dynamic PET/MR imaging over a period of two hours. At the end of the experiment the mice were sacrificed to allow HPLC analysis for radiolabeled metabolites of plasma and urine samples. Selected organs were withdrawn and checked for radioactivity.

**Results:** Even though one of the investigated compounds, [18F]2-fluoroethylazide (18F-FEAz), was reported by Evans et al. [1] to show promising properties to be used as PDR, the conducted experiments indicated rapid metabolization and high bone uptake. Metabolite analysis confirmed degradation and formation of free 18F-fluoride. Hence, aiming for improved metabolic stability, structural modifications led to the compounds 18F-FPAz and 18F-2-FPA, which were also labeled in excellent yields. Both novel compounds are highly valuable as prosthetic groups for “click” radiolabeling due to improved accessibility of cold reference materials (as compared to FEAz). Unfortunately 18F-FPAz and 18F-2-FPA did not show improved in vivo stability, thus limiting their use as in vivo click agent (PDR) for pretargeted PET imaging.

**Conclusion:** In the course of this work we were able to prepare a novel set of [18F]-fluoroalkylazides, whose cold reference materials exhibit favorable physical properties and accessibility compared to already reported [18F]2-fluoroethylazide. Even though our studies showed that the synthesized compounds are not suitable for in vivo imaging, they seem to be attractive candidates for application in rapid [18F]-radiolabeling, which is currently under investigation.

**References**

[1] Evans, H. L., Slade, R. L., Carroll, L., Smith, G., Nguyen, Q., Iddon, L., Kamaly, N., Stöckmann, H., Leeper, F. J., Aboagyea, E. O., Spiveyc, A.C., Copper-free click - a promising tool for pre-targeted PET imaging, Chem. Commun., 2012, 48, 991–993.

## A21 Microfluidic 68Ga-radiolabeling of PSMA-HBED-CC using a flow-through reactor

### S Pfaff^1,2^, C Philippe^1^, M Mitterhauser^1,3^, M Hartenbach^1^, M Hacker^1^, W Wadsak^1,2^

#### ^1^Department of Biomedical Imaging and Image-guided Therapy, Division of Nuclear Medicine, Medical University of Vienna, Vienna, Austria; ^2^Department of Inorganic Chemistry, University of Vienna, Vienna, Austria; ^3^LBI for Applied Diagnostics, Vienna, Austria

**Aim:** In radiochemistry, main focus is set on time efficiency, automated syntheses as well as high chemical conversion. To meet these requirements, microfluidic devices have been tested for downsizing the precursor amount of already established radiosyntheses during the last years [1]. This approach usually enables an enhanced conversion due to a high surface-to-volume ratio [2]. Microfluidic devices have scarcely been tested for Ga-68-radiolabeling so far [3]. The aim of this study was to test the feasibility of using a fully automated microfluidic system containing a flow-through reactor for the radiosynthesis of Ga-68-PSMA-HBED-CC.

**Methods:** Ga-68-radiolabeling of PSMA-HBED-CC (ABX, GMP grade) was performed using an Advion NanoTek LF system containing a flow-through microreactor that consists of a silica capillary (l = 2 m, Ø100 μm, V = 15.6 μL). 68Ga3+ was obtained from a Ge-68/Ga-68 generator (3.7 GBq; Obninsk) and the eluate was collected according to a fractionized protocol. Roughly 400 μL of the peak activity were loaded into the storage loop within the NanoTek system and aliquots of different volumes were used for subsequent reactions. For optimization of PSMA-HBED-CC radiolabeling different flowrates (i.e. 30, 50, 80 μL/min), reaction volumes (40–740 μL), temperatures (25-150 °C) and precursor concentrations (0.2-3 nmol) were evaluated. NaOAc- and HEPES-solutions (1.5 M) were used for pH adjustment.

**Results:** Ga-68-radiolabeling of PSMA-HBED-CC (ABX, GMP grade) was performed using an Advion NanoTek LF system containing a flow-through microreactor that consists of a silica capillary (l = 2 m, Ø100 μm, V = 15.6 μL). 68Ga3+ was obtained from a Ge-68/Ga-68 generator (3.7 GBq; Obninsk) and the eluate was collected according to a fractionized protocol. Roughly 400 μL of the peak activity were loaded into the storage loop within the NanoTek system and aliquots of different volumes were used for subsequent reactions. For optimization of PSMA-HBED-CC radiolabeling different flowrates (i.e. 30, 50, 80 μL/min), reaction volumes (40–740 μL), temperatures (25-150 °C) and precursor concentrations (0.2-3 nmol) were evaluated. NaOAc- and HEPES-solutions (1.5 M) were used for pH adjustment.

**Conclusion:** To the best of our knowledge, we were able to demonstrate for the first time the successful microfluidic Ga-68-radiolabeling of PSMA-HBED-CC. Moreover, we observed that radiochemical conversion can be further enhanced, when purging the system with all reagents prior to syntheses. This proof-of-principle might lead to increased availability of Ga-68-tracers following the dose-on-demand principle.

**References**

(1) Lee et al., Science 2005; 310: 1793–1796

(2) Ungersboeck et al., Nuc Med Bio 2012; 39: 1087–1092

(3) Zeng et al., Nuc Med Bio 2013; 40: 42–51.

## A22 Influence of 24-nor-ursodeoxycholic acid on hepatic disposition of [18F]ciprofloxacin measured with positron emission tomography

### T Wanek^1^, E Halilbasic^2^, M Visentin^3^, S Mairinger^1^, B Stieger^3^, C Kuntner^1^, M Trauner^2^, O Langer^1,4^

#### ^1^Health and Environment Department, Biomedical Systems, AIT Austrian Institute of Technology GmbH, Seibersdorf, Austria; ^2^Division of Gastroenterology and Hepatology, Department of Internal Medicine III, Medical University of Vienna, Vienna, Austria; ^3^Department of Clinical Pharmacology and Toxicology, University Hospital, Zurich, Switzerland; ^4^Department of Clinical Pharmacology, Medical University of Vienna, Vienna, Austria

**Aim:** 24-nor-ursodeoxycholic acid (norUDCA) is a novel therapeutic approach to cholestatic liver diseases. In mouse models of cholestasis norUDCA induces basolateral multidrug resistance-associated proteins 4 and 3 (Mrp4, Mrp3) in hepatocytes, which provide alternative escape routes for bile acids accumulating during cholestasis but could also result in altered hepatic disposition of concomitantly administered substrate drugs.

**Methods:** We used PET imaging to study the influence of norUDCA on hepatic disposition of the model Mrp4 substrate [18F]ciprofloxacin in wild-type and Mdr2(−/−) mice, a model of cholestasis. Animals underwent 90 min dynamic [18F]ciprofloxacin PET scans at baseline and after 5 days of oral norUDCA treatment.

**Results:** Following norUDCA treatment, liver-to-blood area under the curve ratio of [18F]ciprofloxacin was significantly decreased compared to baseline, both in wild-type (−34.0 ± 2.1 %) and Mdr2(−/−) mice (−20.5 ± 6.0 %). [18F]Ciprofloxacin uptake clearance from blood into liver was reduced by −17.1 ± 9.0 % in wild-type and by −20.1 ± 7.3 % in Mdr2(−/−) mice. Real-time PCR analysis showed significant increases in hepatic Mrp4 and Mrp3 mRNA following norUDCA. Transport experiments in OATP1B1-, OATP1B3- and OATP2B1-transfected cells revealed weak transport of [14C]ciprofloxacin by OATP1B3 and OATP2B1 and no inhibition by norUDCA.

**Conclusion:** Our data suggest that changes in hepatic [18F]ciprofloxacin disposition in mice following norUDCA treatment were caused by induction of basolateral Mrp4 in hepatocytes. If norUDCA also induces basolateral ABC transporters in humans, this could potentially lead to transporter-mediated drug-drug interactions for drugs which are substrates of these transporters and for which the liver is the main clearance and/or therapeutic target organ. Finally, we speculate that [18F]ciprofloxacin PET may allow to functionally visualize the pharmacologically induced adaptive basolateral ABC transporter response in cholestatic disorders.

## A23 Automated 18F-flumazenil production using chemically resistant disposable cassettes

### P Lam^1^, M Aistleitner^1^, R Eichinger^1^, C Artner

#### ^1^IASON GmbH, Feldkirchnerstraße 4, A-8054 Graz-Seiersbergm, Austria

**Aim:** Production of 18F-flumazenil from 2-nitroflumazenil under GMP conditions is primarily performed with manipulators or custom built radiosynthesis modules with fixed components, as the use of N,N-dimethylformamide (DMF) at 160 °C prohibits the use of commonly available disposable cassettes. Cassettes for the GE TRACERlab MXFDG synthesis modules are commercially available in different materials. ROTEM Industries and GE Healthcare use polysulfone as the main component, and it is known that this polymer is not compatible with DMF. Cassettes from ABX are reportedly more chemical resistant, with polypropylene as its main constituent. The aim of this work was to establish a robust automated radiosynthesis procedure for 18F-flumazenil on the GE TRACERlab MXFDG, where staff previously trained on the same equipment can easily perform the radiosynthesis.

**Methods:** Fragments of the ABX FDG cassette was exposed for 1 or 24 h to various solvents including acetonitrile and DMF at room temperature (RT) or 60 °C to assess its chemical resistance. A new synthesis sequence was written for the GE TRACERlab MXFDG. A non-radioactive test run was performed with the ABX FDG cassette, and radiosynthesis was carried out with the cassette components replaced by chemically resistance manifolds (ABX).

**Results:** Incubation of the PP ABX FDG cassette fragments in acetonitrile at RT showed no macroscopic influence on the polymer while the fragment became soft and mouldable at 60 °C. Visible changes occurred after 1 h incubation with DMF, with the polymer having completely dissolved after 24 h, regardless of temperature. To verify the implication of these results on a 1 h radiosynthesis, a test run was performed using the same cassette material and synthesis conditions without radioactivity. Parts of the manifold was found to have cracked, and a valve blockade was observed due to polymer melting. Radiosynthesis was carried out with the manifolds replaced by chemically resistant ones, and 18F-flumazenil was successfully obtained in 4 % decay corrected yield.

**Conclusion:** Disposable cassettes assembled with chemically resistant manifolds is a requirement for the automated synthesis of 18F-flumazenil from 2-nitroflumazenil using the GE TRACERlab MXFDG synthesis module. Neither reagent kits nor program sequences are hitherto commercially available, but GMP grade production can nonetheless be successfully carried out with modifications on existing equipment.

## A24 Similarities and differences in the synthesis and quality control of 177Lu-DOTA-TATE, 177Lu -HA-DOTA-TATE and 177Lu-DOTA-PSMA (PSMA-617)

### H Eidherr^1^, C Vraka^1,2^, A Haug^1^, M Mitterhauser^1,3^, L Nics^1,2^, M Hartenbach^1^, M Hacker^1^, W Wadsak^1^

#### ^1^Department of Biomedical Imaging and Image-guided Therapy, Division of Nuclear Medicine, Medical University of Vienna, Vienna, Austria; ^2^Department of Nutritional Sciences, Faculty of Life Sciences, University of Vienna, Vienna, Austria; ^3^LBI for Applied Diagnostics, Vienna, Austria

**Aim:** Over the last years, peptide receptor radiotherapy (PRRT) using 177Lu in the treatment of neuroendocrine tumours (NET) and prostate cancer (PCa) has become a major issue in nuclear medicine. Consequently, the safe and reliable production and quality control of therapeutic amounts of suitable radiopharmaceuticals, i.e. 177Lu-DOTA-TATE and 177Lu-HA-DOTA-TATE (“high affinity DOTA-TATE”) for NET-treatment as well as 177Lu-DOTA-PSMA (PSMA-617) against PCa, had to be established and optimized. Similarities and differences in the set-up are reported in this work.

**Methods:** 177Lu-radiolabelling was performed using the cassette-based ModularLab PharmTracer® synthesizer (EZAG, Berlin, Germany) and starting activities of 9.0-19.0GBq 177LuCl3 (no-carrier-added; itg, Munich, Germany) in 0.5 ml 0.04 M HCl. For the somatostatin analogues, approximately 250-500 μg of DOTA-TATE (ABX, Radeberg, Germany) or HA-DOTA-TATE (PiChem, Graz, Austria) – calculated as 1 μg peptide/40 MBq 177LuCl3 + 10 % peptide, dissolved in deionized water – were reacted at 80 °C/20 min in 0.57 M ascorbate buffer, pH 4.5. For DOTA-PSMA (ABX), approximately 130-270 μg of DOTA-PSMA – 1 μg peptide/65 MBq 177LuCl3 peptide, dissolved in Ethanol (48 %) – was reacted at 90 °C/10 min in 0.57 M ascorbate buffer, pH 4.5 and with 200 μl Ethanol (96 %). All precursors were of GMP quality. Crude 177Lu-DOTA-TATE/-HA-DOTA-TATE was purified on a C18 SPE cartridge without any further dilution, whereas 177Lu-DOTA-PSMA was first prediluted with 2 ml saline (0.9 %), before purification on a C18-SepPak by washing with 2 ml saline (0.9 %). Elution was done with 2 ml ethanol (48 %) and the product was transferred and 0.22 μm-filtrated to the product vial together with 16 ml saline (0.9 %). To all 177Lu-DOTA-peptide-formulations 0.5-1.2 ml DTPA (3 mg/ml) was added. Full radiopharmaceutical quality control was performed for all 177Lu-peptide formulations (e.g. radio-TLC-, HPLC-, pH-, sterility- and apyrogenicity testing).

**Results:** 177Lu-labelling of DOTA-coupled somatostatin analogues was introduced at the Medical University / General Hospital of Vienna in 2010 and 177Lu-DOTA-TATE- syntheses were performed until 2015 (n = 109). Due to legal restrictions in the availability of DOTA-TATE precursor we recently switched to the use of 177Lu-HA-DOTA-TATE (n = 13, so far). Also this year, the production of 177Lu-DOTA-PSMA was implemented (n = 14, so far). All 177Lu-productions took about 45 min (synthesis, including conditioning, purification and sterile filtration) plus 120 min for preparation of the devices plus 60 min for QC. The synthesis failure rate was 99 %. All 177Lu-lots were sterile and endotoxins were <1EU/ml. The HA-DOTA-TATE-precursor was somewhat less soluble than DOTA-TATE. 177Lu-DOTA-PSMA showed a slightly different chromatographic behaviour in TLC and HPLC compared to 177Lu-DOTA-TATE and 177Lu-HA-DOTA-TATE.

**Conclusion:** Both the somatostatin analogues 177Lu-DOTA-TATE and 177Lu-HA-DOTA-TATE for NET-treatment but also 177Lu-DOTA-PSMA for treatment of PCa could be safely produced in activities up to <16GBq (2 therapeutic doses) and excellent quality under observation of proper radioprotection and radioactive waste management. Differences in the preparations were of minor concern. Set-up for the 3 different radiotherapeutics could be easily adapted.

## A25 68Ga- and 177Lu-labelling of PSMA-617

### H Kvaternik^1^, R Müller^2^, D Hausberger^1^, C Zink^1^, RM Aigner^1^

#### ^1^Department of Radiology, Division of Nuclear Medicine, Medical University of Graz, Graz, Austria; ^2^ Seibersdorf Labor GmbH, Austria

**Aim:** PSMA is a most promising ligand for imaging and therapy of prostate cancer lesions. Ever since the DOTA conjugated ligand PSMA-617 is known, the DOTA ligand can be labelled with a broad range of radionuclides (1). Our aim was, on the one hand, to establish labelling of 68Ga-PSMA-617 for PET diagnosis, and on the contrary 177Lu-PSMA-617 for therapy. Both should be compliant to the GMP preparation methodology.

**Methods:** The ligand PSMA-617 was purchased from ABX. 68Ga-PSMA-617 was prepared with a cationic purified (5 M NaCl, SCX PS-H+) 68GaCl3 (1.8 GBq Generator, IThemba). The labelling of 20 μg PSMA-617 was performed in HEPES solution at 125 °C/6 min. The crude mixture was purified by C18 SPE. For 177Lu-PSMA-617, 200 μg PSMA-617 was labelled with 10 GBq 177LuCl3 (ITG) in sodium acetate/ascorbate buffer at 100 °C/20 min. Then, the reaction mixture was diluted and sterile filtrated without further purification. Both kinds of synthesis was carried out with a Scintomics GRP synthesizer, sterile single use cassettes and the standard synthesis sequences for 68Ga-peptides respective therapeutic peptides.

**Results:** 68Ga-PSMA-617 was obtained with a yield of 48 % at EOS (decay corr. 79 %). The labelling reaction was near quantitative, but 15 % (decay corr.) of 68Ga retained on the cationic exchange column during processing. The labelling to 177Lu-PSMA-617 was quantitative, there for the yield was >90 %. The radiochemical purity, determined to be HPLC was 96 % (68Ga-PSMA-617) and 92 % (177Lu-PSMA-617) due to detected impurities of labelled peptides. The detected amount of free radionuclides were always <1 %. 68Ga-PSMA-617 and 177Lu-PSMA-617 remained stable until the planned shelf live.

**Conclusion:** The introduced preparation of 68Ga-PSMA-617 and 177Lu-PSMA-617 using Scintomics GRP synthesizer and standard synthesis sequences are robust and reliable processes, which meets the GMP rules.

**References**

(1) Afshar-Oromieh A, et al. J Nucl Med. 2015 Aug 20. doi: 10.2967 / jnumed.115.16.1299

## A26 Radiolabelling of liposomes with 67Ga and biodistribution studies after administration by an aerosol inhalation system

### U Cossío^1^, M Asensio^2^, A Montes^2^, S Akhtar^3^, Y te Welscher^3^, R van Nostrum^3^, V Gómez-Vallejo^1^, J Llop^1^

#### ^1^CIC biomaGUNE, Edificio Empresarial "C", Paseo de Miramón 182, 20009, Donostia, Spain; ^2^Engineering Department, Ingeniatrics Tecnologies, P.I. Parque Plata, Camino Mozárabe 41, 41900, Camas-Sevilla, Spain; ^3^Department of Pharmaceutics, University of Utrecht, Utrecht, The Netherlands

**Aim:** Liposomes (LPs) have been extensively investigated as nanocarriers for drug delivery. In this context, evaluation of their biodistribution pattern after administration into living organisms is of paramount importance. This can be achieved by incorporation of a γ-emitter radioisotope followed by in vivo imaging using single photon emission computed tomography (SPECT). Here, we present a method for radiolabelling LPs with the γ-emitter 67Ga. Regional distribution in the lungs after inhalation using a specially designed nebulisation chamber was determined using SPECT-CT.

**Methods:** Gallium-67 (T1/2 = 3.26 days) was purchased from IBA-Molypharma as 67Ga-citrate complex and converted into 67GaCl3 following a well established method.1 For the radiolabelling, 10 μL 8-hydroxyquinoline (10 mM in EtOH) in 100 μL sodium acetate buffer (100 mM pH 5.5, 150 mM NaCl) were added to 67GaCl3 (≈150 MBq in 0.1 M HCl). Immediately after, 100 μL of DTPA-containing LPs were added and the mixture was incubated at 37 °C for 1 hour. 67Ga-liposomes were purified by size exclusion chromatography and radiochemical purity was assessed by radio-ITLC. Administration of the radiolabelled LPs was carried out under sedation using an only-nose exposition device coupled to a chamber equipped with a specially designed nebulizer. Biodistribution of 67Ga-liposomes was investigated by whole body SPECT-CT imaging. Dissection/gamma counting and ex vivo imaging experiments to assess the percentage of the nebulised dose deposited in the lungs and the accurate regional distribution, respectively, were also carried out.

**Results:** Labelling efficiencies close to 45 % were achieved. After purification, radiochemical purity exceeded 95 % in all cases. Biodistribution data showed a uniform distribution of the labelled LPs in both lungs at short times after inhalation. The percentage of the nebulised LPs reaching the lungs was close to 0.5 %.

**Conclusion:** Uniform distribution of the labelled LPs in the lungs is achieved with the new aerosol generator. The obtained data constitute a promising starting point for using liposomes as nanocarriers for antibiotics targeting the lungs of infected patients.

**References**

1. V. Ščasnár, J. Lier, European Journal of Nuclear Medicine, 1993, 20, 273

## A27 Fully automated quantification of DaTscan SPECT: Integration of age and gender differences

### F VandeVyver, T Barclay, N Lippens, M Troch

#### AZ St-Lucas Gent, Belgium

**Aim:** SPECT imaging of dopamine transporters (DaT) with FP-CIT (DaTscan) is an established method for the diagnosis of neurodegenerative parkinsonism. Quantification of DaTscan (semi-automatic or full automatic) has been shown to reduce equivocal reporting of DaTscan and can be used as an adjunct to visual assessment. The recent published “European multicentre database of healthy controls” [1] showed significant age-related effects and gender differences in DaT levels in healthy controls. We developed a tool to integrate those normal physiological variances in the quantification of a single SPECT-examination. Furthermore the tool allows to build a large database for further statistical analysis.

**Methods:** SPECT-acquisition was done on a GE Hawkeye dual head gamma-camera. Quality control showed longitudinal uniformity and centre of rotation within limits. SPECT acquisition and reconstruction were performed in concordance with the parameters described in the ENC-DAT study. Images were reconstructed without and with attenuation correction (using Chang’s method). For further calculation only the results of the AC images were used. Quantification was done using BRASS fully automated functional brain analysis (Hermes, Sweden).

Using standard Excel software (Microsoft Corporation, United States) a macro enabled worksheet was developped which 1) allows easy input of relevant parameters (including quantitative output of BRASS) for an individual patient, 2) create a result page with numerical and graphical results of the DaTscan-quantification and 3) builds a database from each input session.

**Results:** In all patients (age ranging from 32 to 87 years; 31 male and 30 female patients) scheduled for DaTscan, quantification was performed with age and gender correction. The procedure is standardised and fully automatic and autonomously done by the medical imager. Physicians can report DaTscan with greater diagnostic confidence: all scans could be classified as normal or abnormal. A database of 61 patients is build since start of this tool: further statistical analysis will be performed in the future.

**Conclusion:** Integreation of age and gender differences in quantification of DaTscan is feasible and applicable in clinical routine.

**References**

1. Varrone A. et al. European multicentre database of healthy controls for [123I] FP-CIT SPECT (ENC-DAT): age-related effects, gender differences and evaluation of different methods of analysis. Eur J Nucl Med Mol Imaging (2013) 40:213–227.

## A28 Lesion-to-background ratio in co-registered 18F-FET PET/MR imaging – is it a valuable tool to differentiate between low grade and high grade brain tumor?

### L Hehenwarter, B Egger, J Holzmannhofer, M Rodrigues-Radischat, C Pirich

#### Department of Nuclear Medicine and Endocrinology, University Hospital Salzburg, Paracelsus Private Medical University Salzburg, Salzburg, Germany

**Aim:** To validate the cut-off value for the differentiation between low grade and high grade glioma in patients undergoing O-(2-18F-fluoroethyl)-L-tyrosine (18F-FET) PET/CT combined with MR imaging.

**Methods:** 232 Lesions (from 112 patients, mean age 54 years) were included into this study. The tumor entities are astrozytoma (45), ependymoma (2), ganglioma (1), glioblastoma (120), gliosarkoma (1), oligoastrozytoma (32), oligodendroglioma (29) and undefined (2). The Philips software Intellispace Portal (ISP) was used to co-register 18F-FET PET data with most recent MRT images with a maximum interval of 42 days. A circular background area with 5 cm diameter drawn in the contra lateral hemisphere to glioma including white and grey matter was suitable to define a LBR of 1,6 as threshold between tumor and normal tissue. For calculating the lesion-to-background ratio (LBR), the SUV max of the lesion was divided by the SUV mean of the background area. Clinical data on surgery, histology, chemotherapy, radiotherapy and WHO grade were obtained from all patients.

**Results:** The mean LBR of low grade tumor was significantly lower compared with the LBR of high grade tumor (2,38 vs. 2,83 p = 0,006). The 95 % confidence interval ranged from 2,067 to 2,686 in low grade tumors, and from 2,695 to 2,967 in high grade tumors, respectively. The LBR cut-off value to differentiate between low grade and high grade tumors was 2,7.

The mean LBR in patients without radiotherapy was not significantly different compared to patients undergoing the 18F-FET PET scan during or after radiotherapy (2,78 vs. 2,70 p = 0,560).

The mean LBR in patients without chemotherapy was significantly higher than in patients undergoing the 18F-FET PET scan during or after chemotherapy (2,93 vs. 2,61 p = 0,023).

**Conclusion:** This study confirms the validity of a cut-off LBR value of 2,7 for the differentiation of low grade and high grade glioma.

**References**

1. Hutterer M, Nowosielski M, Putzer D, et al. [18F]-fluoro-ethyl-L-tyrosine PET: a valuable diagnostic tool in neuro-oncology, but not all that glitters is glioma. Neuro-Oncology 2013; 15(3):341–351

2. Pauleit D, Floeth F, Hamacher K, et al. O-(2-[18F]fluoroethyl)-L-tyrosine PET combined with MRI improves the diagnostic assessment of cerebral gliomas. Brain 2005; 128:678–687

3. Rapp M, Heinzel A, Galldiks N, et al. Diagnostic Performance of 18F-FET PET in Newly Diagnosed Cerebral Lesions Suggestive of Glioma. J Nucl Med 2013; 54:229–235

4. Langen K, Bartenstein P, Boecker H, et al. PET- und SPECT-Untersuchungen von Patienten mit zerebralen Gliomen mittels radioaktiv markierter Aminosäuren. DGN-Handlungsempfehlungen 2013

## A29 [11C]-methionine PET in gliomas - a retrospective data analysis of 166 patients

### N Pötsch^1^, I Rausch^2^, D Wilhelm^1^, M Weber^1^, J Furtner^1^, G Karanikas^1^, A Wöhrer^3^, M Mitterhauser^1^, M Hacker^1^, T Traub-Weidinger^1^

#### ^1^Department of Biomedical Imaging and Image-guided Therapy, Medical University of Vienna, Vienna, Austria ;^2^Center for Medical Physics and Biomedical Engineering, Medical University of Vienna, Vienna, Austria; ^3^Institute of Neurology, Medical University of Vienna, Vienna, Austria

**Aim:** To evaluate visually and semiquantitatively [11C]-methionine (MET) PET and MRI for glioma grading in non treated patients.

**Methods:** A total of 166 glioma patients (93 men, 73 women, mean age: 44.9, range 18–83.5 years) were included in this study. Patients underwent a preoperative MET PET and MRI imaging between 2000 and 2014 at the Medical University of Vienna. MRI images were evaluated for contrast agent enhancement of the tumor. The PET scans were evaluated visually and by means of SUV tumor to background (T/N) ratios extracted from a cubic volume of interest (8 cm3). Following T/N ratios were evaluated: T/Nstandard (i.e. SUVmax/SUVmean), T/Npeak (i.e. SUVpeak/SUVmean), T/Nmax (i.e. SUVmax/ SUVmax) and T/Npp (i.e. SUVpeak/SUVpeak).

**Results:** Our patient contingent was evenly distributed between low and high grade tumors (46 % low, 54 % high grade) according to the latest WHO definition (2007). Excluding oligodendrogliomas (ODG) 61 % of all gliomas without MRI contrast enhancement were low grade and 76 % of gliomas with MRI contrast enhancement were high grade gliomas. In ODGs only 58.5 % of non contrast agent avid gliomas were low grade and 54.5 % with verifiable uptake of contrast agent were high grade gliomas. Most frequently tumors were located in the frontal (44 %) followed by the temporal lobe (41 %). Visually negative PET scans were predominantly found in low grade gliomas (82 % of cases). Focal pattern with a branch and multifocal with branches were mainly found in high grade gliomas (78 %, 70 % of cases). All T/N ratios showed significant results for distinguishing low from high grade gliomas (p < 0.001). T/Nmax and T/Nstandard performed similar and slightly better than T/Npeak and T/Npp when evaluated by AUC of the ROC analysis. Excluding ODGs thresholds of 1.26 T/Nmax and 2.16 T/Nstandard appeared as most applicable (T/Nmax: detection rate 86 %; error rate 31 % resp. T/Nstandard: detection rate 82 %; error rate 22 %).

**Conclusion:** [11C]-methionine PET is useful to distinguish between low and high grade gliomas in non treated patients.

## A30 18F-Fluorocholine versus 18F-Fluorodeoxyglucose for PET/CT imaging in patients with relapsed or progressive multiple myeloma: a pilot study

### T Cassou-Mounat^1,2^, S Balogova^2,3^, V Nataf^4^, M Calzada^1^, V Huchet^2^, K Kerrou^2^, J-Y Devaux^1^, M Mohty^5,6,7^, L Garderet^5^, J-N Talbot^2^

#### ^1^Department of Nuclear Medicine, Hôpital Saint Antoine, AP-HP et Université Pierre et Marie Curie (UPMC), Paris, France; ^2^Department of Nuclear Medicine, Hôpital Tenon, AP-HP & Université Pierre et Marie Curie (UPMC), Paris, France; ^3^Department of Nuclear Medicine, Comenius university & St. Elisabeth Oncology Institute, Bratislava, Slovakia; ^4^Radiopharmacy, Hôpital Tenon, AP-HP, Paris, France; ^5^Hematology, Université Pierre et Marie Curie, Paris, France; ^6^Hôpital Saint-Antoine, AP-HP, Paris, France; ^7^INSERM UMRs U938, Paris, France

**Aim:** PET/CT is increasingly used to evaluate multiple myeloma (MM) bone lesions, however, 18F-fluorodeoxyglucose (FDG), the reference radiopharmaceutical in oncology, shows in this indication relatively low sensitivity. The aim of our pilot study was to compare FDG and 18F-fluorocholine (FCH) in detection of MM lesions at time of disease relapse or progression.

**Methods:** This retrospective study assessed the results of FDG and FCH imaging in 17 MM patients undergoing PET/CT for suspected relapse or progressive MM. For each patient and each tracer, an on-site reader and a blinded reader independently determined the number of foci of tracer uptake in bone and the intensity of up to 5 foci as given by their SUVmax and the corresponding target/non-target ratio (T/NT).

**Results:** The blinded reader detected 63 foci for FDG versus 103 foci for FCH (+64 %) whereas the on-site reader detected 66 foci for FDG versus 102 foci for FCH (+55 %), the difference being significant in each case (P = 0.002 and 0.008, respectively). Between-reader agreement on the total number of foci was very high, with a kappa coefficient of 0.83 (CI 0.746-0.940) for FDG and an even higher coefficient for FCH (0.93, CI 0.870-0.995). Measurement of uptake by the blinded reader in the 54 foci that took up both tracers revealed a significantly higher median SUVmax for FCH versus FDG [6.10, range (2.3-16); vs 4.65, range (1–20.6); P = 0.0007).

**Conclusion:** These findings suggest that PET/CT performed for suspected relapsing or progressive MM would reveal more lesions when using FCH rather than FDG.

## A31 Prognostic benefit of additional SPECT/CT in sentinel lymph node mapping of breast cancer patients

### S Stanzel^1^, G Pregartner^2^, T Schwarz^1^, V Bjelic-Radisic^3^, B Liegl-Atzwanger^4^, R Aigner^1^

#### ^1^ Medical University of Graz, Department of Radiology, Division of Nuclear Medicine, Graz, Austra; ^2^Medical University of Graz, Institute for Medical Informatics, Statistics and Documentation, Graz, Austria; ^3^Medical University of Graz, Department of Gynecology and Obstetrics, Graz, Austria; ^4^Medical University of Graz, Institute of Pathology, Graz, Austria

**Aim:** The objective of the study was to demonstrate the advantages of SPECT/CT-aided sentinel lymph node mapping (SLNM) on metastatic node detection and disease-free survival in patients with invasive breast cancer.

**Methods:** Two hundred thirty-two patients with invasive breast cancer with clinically negative lymph nodes were included in this study as they were referred for SLNM with 99mTc-nanocolloid. Of those patients, 118 had planar scintigraphy alone, and 114 had planar scintigraphy and low-dose SPECT/CT additionally.

**Results:** Between April 2002 and June 2011, 118 patients underwent the standard SLNM technique. Between December 2009 and December 2011, 114 patients underwent the SPECT/CT technique. In the SPECT/CT cohort, 139 SLNs were detected with planar scintigraphy alone and 290 SLNs with additional SPECT/CT. In 5 patients, no SLN could be detected with planar scintigraphy. In 4 of the last patients, SLNs were identified with the aid of SPECT/CT. In the standard cohort, only 125 SLNs were detected. Thus, in the SPECT/CT cohort significantly more SLNs per patient were removed than in the standard cohort (2.25 vs. 1.89), and the number of further on identified metastatic SLNs per patient was significantly higher (0.43 vs. 0.31). Consequently, in the SPECT/CT cohort significantly more patients had therapy change (axillary lymph node dissection) than in the standard cohort (32 vs. 15) and the local relapse/metastasis rate in the SPECT/CT cohort was lower than in the standard cohort (8.8 % vs. 26.3 %). Accordingly, 4-year disease-free survival was significantly longer in the SPECT/CT cohort (91 % vs. 74 %).

**Conclusion:** Among patients with breast cancer, the use of SPECT/CT-aided SLNM correlated due to a better anatomical localization and identification of planar not visible SLNs with a higher detection rate of metastatic SLNs. This led to therapeutic consequences and, therefore, a higher rate of disease-free survival.

**References**

1. Buscombe J, Paganelli G, Burak ZE, et al. Sentinel node in breast cancer procedural guidelines. Eur J Nucl Med Mol Imaging 2007; 34:2154–2159

2. von Forstner, C. Der Einsatz von SPECT/CT bei der Wächterlymphknotendiagnostik. Der Nuklearmediziner 2011; 34:59–65.

3. Kuehn T, Bembenek A, Decker T, et al. Consensus Committee of the German Society of Senology. A concept for the clinical implementation of sentinel lymph node biopsy in patients with breast carcinoma with special regard to quality assurance. Cancer 2005; 103:451–461

4. Gallowitsch HJ, Kraschl P, Igerc I, et al. Sentinel node SPECT-CT in breast cancer. Can we expect any additional and clinically relevant information? Nuklearmedizin 2007; 46:252–256.

5. van der Ploeg IM, Valdés Olmos RA, Nieweg OE, Rutgers EJ, Kroon BB, Hoefnagel CA. The additional value of SPECT/CT in lymphatic mapping in breast cancer and melanoma. J Nucl Med 2007; 48:1756–1760

6. van der Ploeg IM, Valdés Olmos RA, Kroon BB, Nieweg OE. The Hybrid SPECT/CT as an additional lymphatic mapping tool in patients with breast cancer. World J Surg 2008; 32:1930–1934

7. Vercellino L, Ohnona J, Groheux D, et al. Role of SPECT/CT in sentinel lymph node detection in patients with breast cancer. Clin Nucl Med 2014; 39:431–436.8. Bennie G, Vorster M, Buscombe J, Sathekge M. The added value of a single-photon emission computed tomography-computed tomography in sentinel lymph node mapping in patients with breast cancer and malignant melanoma. World J Nucl Med. 2015; 14:41–46.

## A32 Evaluation of diagnostic value of TOF-18F-FDG PET/CT in patients with suspected pancreatic cancer

### S Stanzel^1^, F Quehenberger^2^, RM Aigner^1^

#### ^1^Medical University of Graz, Department of Radiology, Division of Nuclear Medicine, Graz, Austria; ^2^Institute for Medical Informatics, Statistics, and Documentation, Graz, Austria

**Aim:** Evaluation of the advantages of time-of-flight (TOF) in 18F-FDG-PET/CT in the detection and characterization of pancreatic lesions in patients with suspected pancreatic cancer. Furthermore, the maximum standard uptake value (SUVmax) was assessed. TOF should improve image noise, local resolution and thus the demarcation of small lesions.

**Methods:** TOF-18F-FDG PET/CT was prospectively performed in 20 patients with suspected pancreatic cancer. In all patients, a histopathologic confirmation was made. PET/CT images were performed 30 and 90 min. p.i. including a diagnostic CT of the upper abdomen with contrast medium and pancreas protocol. SUVmax of the lesions was measured and compared with and without TOF in the 30 and 90 min. p.i. images. Lesions with an increase of SUVmax on the delayed images were assessed as malignant, whereas lesions with a decrease of SUVmax were assessed as benign. The comparison of TOF-PET/CT with standard PET/CT images and the determination of cut-off values for malignant lesions were made using ROC-analysis.

**Results:** Of 20 patients 11 patients had malignant and 9 patients a benign pancreatic lesion. In one patient IPMN could only be diagnosed correctly with TOF. Based on the histopathologic findings sensitivity, specificity and diagnostic accuracy for TOF were 100 %, 67 % und 85 % versus 90 %, 90 % and 90 % without TOF. ROC analysis of SUVmax with TOF 30 and 90 min p.i. yielded a cut-off value of 4.5 and 4.7, respectively with a sensitivity and specificity of 89 % and 100 % and 91 % and 100 %, respectively. The area under the curve (AUC)PET and AUCTOF-values showed no significant difference neither in the early nor the delayed images.

**Conclusion:** Among patients with suspected pancreatic cancer, the use of TOF-PET/CT correlated due to a better anatomical localization and identification of pancreatic lesions with higher sensitivity. However, TOF-PET/CT is of no additional use in differentiating malignant and benign pancreatic lesions. To what extent patients can benefit from the higher statistical weighting of the sensitivity should be examined in a greater patient population.

**References**

[1] Reske SN, Kotzerke J. FDG-PET for clinical use. Results of the 3rd German Interdisciplinary Consensus Conference, “Onko-PET III”, 21 July and 19 September 2000. Eur J Nucl Med 2001; 28:1707–23.

[2] Pfannenberg AC, Aschoff P, Brechtel K, Müller M, et al. Value of contrast-enhanced multiphase CT in combined PET/CT protocols for oncological imaging. Br J Radiol 2007; 80:437–445.

[3] Strobel K, Heinrich S, Bhure U, Soyka J, Veit-Haibach P, et al. Contrast-Enhanced 18F-FDG PET/CT: 1-Stop-Shop Imaging for Assessing the Resectability of Pancreatic Cancer. J Nucl Med 2008; 49:1408–1413.

[4] Takanami K, Hiraide T, Tsuda M, Nakamura Y, Kaneta T, et al. Additional value of FDG PET/CT to contrast-enhanced CT in the differentiation between benign and malignant intraductal papillary neoplasms with mural nodules. Ann Nucl Med 2011; 25:501–510.

[5] Orlando LA, Kulasingam SL, Matchar DB. Meta-analysis: the detection of pancreatic malignancy with positron emission tomography. Aliment Pharmacol Ther 2004; 20:1063–70.

[6] Heinrich S, Goerres GW, Schäfer M, Sagmeister M, Bauerfeind P, et al. Positron emission tomography/computed tomography influences on the management of resectable pancreatic cancer and ist cost-effectiveness. Ann Surg 2005; 242:235–43.

[7] Kerschbaumer S., Gstettner C., Aigner R. Evaluation of the clinical impact of Time‐of‐Flight (TOF) PET/CT scans for F18‐FDG whole body protocol on a Siemens Biograph mCT. EANM Scientific Program, Poster Session, Tracking Number 2012-S-1013-EANM.

[8] Hausmann D, Bittencourt LK, Sertdemir M, Weidner A, Büsing K, et al. Evaluation der diagnostischen Genauigkeit der 18F-Cholin PET/CT mit Time-of-Flight-Rekonstruktionsalgorithmus (TOF) bei Prostatakarzinompatienten mit biochemischem Rezidiv. 94. Deutscher Röntgenkongress 2013, Vorträge, VO 310.1, S207-S208.

[9] Werner ME, Karp JS. TOF PET offset calibration from clinical data. Phys Med Biol 2013; 58:4031–46.

[10] Surti S, Scheuermann J, El Fakhri G, Daube-Witherspoon, ME, Lim R et al. Impact of Time-of-Flight PET on Whole-Body Oncologic Studies: A Human Observer Lesion Detection and Localization Study. J Nucl Med 2011; 52:712–719.

[11] Lois C, Jakoby BW, Long MJ, Hubner KF, Barker DW, et al. An Assessment of the Impact of Incorporating Time-of-Flight Information into Clinical PET/CT Imaging. J Nucl Med 2010; 51:237–245.

## A33 New quantification method for diagnosis of primary hyperpatahyroidism lesions and differential diagnosis vs thyropid nodular disease in dynamic scintigraphy

### A Koljević Marković^1^, Milica Janković^2^, V Miler Jerković^2^, M Paskaš^3^, G Pupić^2^, R Džodić^2^, D Popović^2^

#### ^1^Institute of Oncology and Radiology of Serbia, Pasterova 14, 11000 Belgrade, Serbia; ^2^National Cancer Research Center Serbia, University of Belgrade- School of Electrical Engineering, Belgrade, Serbia; ^3^National Cancer Research Center Serbia, Innovation Center, University of Belgrade – Faculty of Electrical Engineering, Belgrade, Serbia

**Aim:** We propose a novel approach additional to inspection and parametric subtraction analysis aiming to avoid thyroid gland and thyroid nodule artifact in parathyroid lesions scintigraphy.

**Methods:** 78 patients, median age of 58 (19–80) years, with clinical diagnosis hyperparathyroidism (PHPT) and ultrasound positive report of concomitant thyroid disease, underwent preoperative, dual- tracer: 99 m Tc-pertecnetat and 99mTc-MIBI, double- phase scintigraphy (EANM guidelines 2009). Specially designed software, we developed, examined ROI time/activity changes in matrix with different, optional sizes, up to maximal resolution (1,5 mm); with automatic imaging signal preprocessing (linear fitted TAC logarithm, ensamble coupling) and segmentation based on semi automatic TACs clustering of pixels as well as fractal analysis for noice/motion artifacts and time activity curves. We correlated our findings with PTH levels, histology results and conventional scintigraphic findings: subtraction and visual interpretation of planar images of neck and mediastinum in oblique sections, according to ultrasound PHPT localization and delayed scans in one hour interval

**Results:** Following histopathology in A.: parathyroid autonomy 53/78pts: solitary- 44pts, hyperplasia: 8pts- 7/8patients with two lesion and 1/7 had all four lesions and one PT carcinoma; median lesion volume was 796 mm^3^. B: thyroid – benign nodule only: adenoma folliculare, colloides and cystica: 25/78(32 %)pts; malignancy: 15/78(19 %)pts- papillary 7pts and one for medullary; C.: concomitant PHPT and thyroid nodular disease occurred in 36/53pts, among them 8pts had thyroid malignancies. Parathormone (PTH) was median 125,2 pg/ml (range 70- 658 pg/ml). Standard findings (subtraction, oblique planar scans and delayed phase) in total of 63 lesion (in 53 pts) had 10 FN (mostly in hypeplasia PTA), and PPV was 81 %; sensitivity 96 %, specificity 76 %. Using specially designed software, thyroid TACs represented exponentially declining curves but parathyroid lesions had typical uptake pattern in the form of late phase peak, independent to PTA volume and/or PTH levels. The ratio of PTA-to-normal thyroid uptake at peak maximum was 1.35(±0.21). Parameters of optimal PHPT curve enabled visual interpretation. Fractal theory was helpful in matrix 5x5 designing classifier dimension 1.1-1.3 for automatic procedure. A new processing method had PPV 97 %, sensitivity 98 %, and specificity 96 %. The thyroid gland washout was up to 28 % in normal, adenoma or thyroid carcinoma tissue and their slope analysis of TACs had equivocal negative slope: −0.04.

**Conclusion:** New quantification method and dedicated software as well as high computational power facilitate the recognition of small parathyroid lesions in the presence of concomitant thyroid nodular disease, overcoming diagnostic problem present in conventional as well as SPECT/CT and PET/CT imaging.

**References**

1. Hindie E, Ugur O, Fuster D et al.”2009 EANM parathyroid guidelines” Eur J Nucl Med Mol Imaging. 2009.36;7:1201–1216.

2. Taillefer R, Boucher Y, Potvin E, Lambert R. Detection and localization of parathyroid adenomas in patients with hyperparathyroidism using a single radionuclide imaging procedure with technetium-99 m-sestamibi (double-phase study). J Nucl Med.1992;33(10):1801–7.

3. O’Doherty MJ, Kettle AG, Wells P, Collins RE, Coakley AJ. Parathyroid imaging with technetium-99 m-sestamibi: preoperative localization and tissue uptake studies. J Nucl Med. 1992;33:313–8.

4. Billotey C, Aurengo A, Najean Y, Sarfati E, Moretti JL, Toubert ME, et al. Identifying abnormal parathyroid glands in the thyroid uptake area using technetium-99 m-sestamibi and factor analysis of dynamic structures. J Nucl Med. 1994;35:1631–6.

5. Blocklet D, Martin P, Schoutens A, Verhas M, Hooghe L, Kinnaert P. Presurgical localization of abnormal parathyroid glands using a single injection of technetium-99 m methoxyisobutylisonitrile: comparison of different techniques including factor analysis of dynamic structures. Eur J Nucl Med.1997;24:46–51.

6. Ansquer C, Mirallie E, Carlier T, Abbey-Huguenin H, Aubron F, Kraeber-Bodere F. Preoperative localization of parathyroid lesions. Value of 99mTc-MIBI tomography and factors influencing detection. Nuklearmedizin.2008;47:158–62.

7. Gomez-Ramirez J, Sancho-Insenser JJ, Pereira JA, Jimeno J, Munne A, Sitges-Serra A. Impact of thyroid nodular disease on 99mTc-sestamibi scintigraphy in patients with primary hyperparathyroidism. Langenbecks Arch Surg.2010;395:929–33.

8. A. Dimitrakopoulou-Strauss, L. Pan, L. G. Strauss. Quantitative approaches of dynamic FDG-PET and PET/CT studies (dPET/CT) for the evaluation of oncological patients. Cancer Imaging.2012;12(1):283–289.

9. Jayender, Jagadeesan, et al. Segmentation of parathyroid tumors from DCE-MRI using Linear Dynamic System analysis. Biomedical Imaging (ISBI).2013 IEEE 10th International Symposium on. IEEE, 2013.

10. Koljević MA, Janković MM, Marković I. et al. Parathyroid dual tracer subtraction scintigraphy: small regions method for quantitative assessment of parathyroid adenoma uptake. Ann Nucl Med.2014.28;8:736–745.

11. Janković, Milica M. et al. GammaKey system for improved diagnostics with gamma cameras. Computers in biology and medicine.2014;50: 97–106.

## A34 A rare case of diffuse pancreatic involvement in patient with merkel cell carcinoma detected by 18F-FDG

### MC Fornito, D Familiari

#### Nuclear Medicine Department and PET/CT center – A.R.N.A.S “ Garibaldi – Nesima”, Via Palermo 636, 95122 Catania, Italy

**Aim:** Merkel cell carcinoma (MCC) is a rare aggressive tumor arising from mechanoreceptors of the epidermis with a lower relative survival stage for stage than in melanoma. Distant metastases are reported in 7 % of the patients with frequently involved sites being distant lymph nodes, liver, lung and brain. Pancreatic involvement is not common and the few cases reported in literature showed one or multiple sites of FDG focal uptake. We report a case of a diffuse involvement of the pancreas detected by 18F-FDG PET-CT in a patient with MCC derived from gluteal skin region.

**Methods:** A 71 years-old male patient with a nodular lesion of about 7 cm on the right gluteus which was excised and diagnosed to be MCC poorly differentiated with Ki67: 80 %. Immuno-histochemical analysis demonstrated positive reaction for neuroendocrine markers such as synaptophysin, CK20, NSE and chromogranin A, while negative for TTF-1 and CK7. Subsequently the patient underwent sentinel lymph node biopsy with final diagnosis of metastasis of MCC in right inguinal station. CT-scan at baseline was negative for distant metastases but, in consideration of the high value of Ki 67 and the lymph nodes metastases, 18F-FDG PET-CT, considered a promising functional imaging modality for the evaluation of MCC, was performed. Patient fasted for at least 6 hours before 18F-FDG administration (blood glucose levels 82 mg/dl) and study was performed using an integrated PET-CT device (Philips Medical System) at 60 minutes from administration of radiotracer intravenously (298 MBq). CT was acquired from the base of the skull to the thighs (120 kV, 50 mA) and used for anatomical localization and attenuation correction of 3D-PET emission data.

**Results:** PET/CT scan showed pathological uptake of the radiotracer into multiple lymph nodes localized in the abdominal and pelvic lymph node stations; in addition the images showed uptake of FDG in a nodular lesion located between right gluteus muscles. The pancreatic gland presented a diffuse and intense uptake of radiotracer (SUV 8.7), without significant morphological changes detected by CT low dose, unlike what is reported in the literature. In according to PET-CT scan the oncologist decided to perform an adjuvant chemotherapy. PET-CT will be performed during and at the end of the treatment for the evaluation of the response.

**Conclusion:** This case confirmes the incremental benefit of PET staging over conventional radiological imaging in the management of MCC patients with unsuspected diffuse pancreas involvement.

**Consent**

Written informed consent was obtained from the patient for publication of this abstract and any accompanying images. A copy of the written consent is available for review by the Editor of this journal.

**References**

1. Iagaru A, Quon A, McDougall IR, Gambhir SS.Merkel cell carcinoma: Is there a role for 2-deoxy-2-[f-18]fluoro-D-glucose-positron emission tomography/computed tomography? Mol imaging boil 2006 Jul-Aug;8(4):212–7

2. Concannon R, Larcos GS, Veness M. The impact of (18)F-FDG PET-CT scanning for staging and management of Merkel cell carcinoma: results from Westmead Hospital, Sydney, Australia. J Am Acad Dermatol. 2010 Jan;62(1):76–84.

## A35 TSH-stimulated 18F-FDG PET/CT in the diagnosis of recurrent/metastatic radioiodine-negative differentiated thyroid carcinomas in patients with various thyroglobuline levels

### P Koranda, H Polzerová, I Metelková, L Henzlová, R Formánek, E Buriánková, M Kamínek

#### Department of Nuclear Medicine, Palacky University and University Hospital, Olomouc, Czech Republic

**Aim:** International thyroid societies recommend 18F-FDG PET/CT in radioiodine-negative thyroid cancer patients with Tg ≥ 10 ng/ml. However according to recent publications, it appears that no clear cutt-off Tg value can be established (1). TSH stimulates thyrocyte metabolism, Glut1 expression in thyroid cells, glucose trapping, and glycolysis and published data suggest that TSH stimulation significantly improves the sensitivity of 18F-FDG-PET/CT (2). The aim of this study was to assess the diagnostic efficiency of TSH-stimulated 18F-FDG PET/CT in radioiodine-negative differentiated thyroid carcinomas (DTCs) with different levels of TSH stimulated thyroglobulin (TSH-Tg).

**Methods:** A total of 109 patients with suspicion of recurrent DTC and negative 131I whole-body scans underwent TSH-stimulated (TSH > 30 mU/L, thyroid hormone withdrawal) contrast-enhanced 18F-FDG PET/CT (400 MBq 18F-FDG/70 kg body weight). 18F-FDG PET /CT was indicated due to elevated TSH-Tg level or according to other suspicions of DTC recurrence (persistently or progressively increased serum TgAb level, high-risk patients, equivocal results of sonography). Patients (pts.) were subdivided into 4 subgroups according to TSH-Tg: Tg < 2 ng/ml (24 pts.), 2 ng/ml < Tg < 10 ng/ml (29 pts.), 10 ng/ml < Tg < 100 ng/ml (42 pts.), and 100 ng/ml < Tg (14 pts.).

**Results:** In the whole group of 109 patients, TSH-stimulated 18F-FDG PET/CT was true positive in 45 cases (41 %), 2 false positive findings (2 %). 18F-FDG PET/CT correctly detected foci of thyroid carcinoma in TSH-Tg subgroups: Tg < 2 ng/ml – 8/24 patients (33 %), 2 ng/ml < Tg < 10 ng/ml – 9/29 patients (31 %), 10 ng/ml < Tg < 100 ng/ml - 19/42 patients (45 %), and 100 ng/ml < Tg - 11/14 patients (79 %). 18F-FDG PET/CT examinations lead to changes in therapeutic strategy (mainly indication of surgery) in 28 patients (26 %).

**Conclusion:** This study shows that TSH-stimulated contrast-enhanced 18F-FDG PET/CT is able to identify radioiodine-negative recurrences or metastases of DTCs in an important part of patients with distinct elevation of TSH-Tg. The detection rate of recurrences is lower in persons with Tg < 10 μg/L or with other reasons of suspicion of metastases (including persistently or progressively increased serum TgAb level), nevertheless the positive findings represent a significant part of these patients. Therefore, this series indicates that it is not possible to determine the lower Tg limit for an indication of TSH-stimulated contrast-enhanced 18F-FDG PET/CT in patients with suspicion of recurrence of differentiated thyroid carcinoma. It could be concluded that TSH-stimulated 18F-FDG PET/CT is an efficient diagnostic tool in DTC patients with a significant impact on therapy.

**References**

1. Salvatori M, Biondi B, Rufini V. 2-[18F]-fluoro-2-deoxy-D-glucose positron emission tomography/computed tomography in differentiated thyroid carcinoma: clinical indications and controversies in diagnosis and follow-up. Eur J Endocrinol 2015; 173(3): R115-130.

2. Ma C, Xie J, Lou Y, Gao Y, Zuo S & Wang X. The role of TSH for 18F-FDG-PET in the diagnosis of recurrence and metastases of differentiated thyroid carcinoma with elevated thyroglobulin and negative scan: a meta-analysis. Eur J Endocrinol 2010; 163: 177–183

## A36 Breast Dose from lactation following I131 treatment

### WH Thomson^1^, C Lewis^2^

#### ^1^Physics and Nuclear Medicine Department City Hospital, Birmingham, UK; ^2^Maternity Department City Hospital, Birmingham, UK

**Aim:** It is well known that breast feeding is totally contra-indicated following I131 treatment for thyrotoxicosis, since I131 is freely expressed in breast milk. However lactation can continue for some time after breastfeeding stops and this can lead to significantly increased breast dose.

**Methods:** ICRP95 developed a comprehensive model for the lactating breast, and breast dose during lactation was calculated. For I131, only one dose model was presented. This was based on normal 30 % uptake by the mother, 270 ml/24 hr milk production, with a further 40 ml milk production stimulated by breast feeding.

**Results:** The breast dose without lactation for 600 MBq I131 (30% uptake) is 36 mSv. The ICRP95 figure with their lactation model is 780 mSv, a 21x increase. Surprisingly, ICRP95 comment that the increased breast dose is small in relation to overall effective dose.

Lactation I131 levels follow plasma levels. So for thyrotoxicosis with an increased thyroid uptake of 55 % the calculated breast dose is 1.2Sv. For ablation with 1.1GBq, the rapid excretion of I131 leads to some breast dose reduction. But calculation suggests a breast dose of 830 mSv, still a high figure.

**Conclusion:** For I131 treatment, the lactation status needs to be considered to reduce breast dose. However suddenly stopping breastfeeding is not recommended and Maternity advice should be sought. Most women can reduce lactation to negligible levels in about 2 weeks with help. In difficult cases, Cabergolin may be considered as an effective mechanism, but can have side effects. Maternity advice is that this is rarely needed. Although uncommon, this situation should be added to local guidance.

**References**

1. “Doses to Infants from Ingestion of Radionuclides in Mothers' Milk.” ICRP Publication 95. Ann. ICRP 34 (3–4), 2004.

## A37 A new concept for performing SeHCAT studies with the gamma camera

### WH Thomson, J O’Brien, G James, A Notghi

#### Physics and Nuclear Medicine Department, City Hospital, Birmingham, UK

**Aim:** SeHCAT absorption studies have gained in popularity over recent years. The capsule has a Se75 activity of only 370 kBq, therefore the gamma camera is normally used intrinsically (no collimators).

However intrinsic counting leaves the crystals exposed, with the potential for anything falling onto the crystal face causing expensive damage. In addition there is the potential for sources, or for any low level (50 kBq) Tc99m contamination on the bed, to interfere with the measurement Some departments count with a LEHR collimator in place but sensitivity is then very low, so counting times are longer and the statistical error can become significant.

**Methods:** A new concept uses a uniform 2 mm lead plate as a crystal cover, or ‘collimator’. This completely blocks any effect from other Tc99m sources in the department. However the higher energy Se75 emissions (264 – 400 keV) can easily penetrate the lead sheet for counting. The lead plate also provides complete protection for the crystal.

**Results:** The sensitivity for Se75 (in a water-filled phantom) with the lead plate is 7608 c/s/MBq. This is 10x the LEHR collimated count rate (GE-LEHR, 766c/s/MBq). Even for a large BMI patient, a 60s count with the lead plate system gives + −0.8 % (2std.dev) error on a 10 % absorption value. However the corresponding error with the LEHR collimator is + −3.5 %. An error of + −1 % would need 1100s counting time for the LEHR collimator.

A point source of Se75 was counted in varying positions and depths in a water-filled phantom. The Variation Index (VI), (average modulus of %differences) was calculated. The more uniform the spread of values then the VI value is smaller. Table 1.

**Conclusion:** The sensitivity for Se75 (in a water-filled phantom) with the lead plate is 7608 c/s/MBq. This is 10x the LEHR collimated count rate (GE-LEHR, 766c/s/MBq). Even for a large BMI patient, a 60s count with the lead plate system gives + −0.8 % (2std.dev) error on a 10 % absorption value. However the corresponding error with the LEHR collimator is + −3.5 % . An error of + −1 % would need 1100s counting time for the LEHR collimator.

A point source of Se75 was counted in varying positions and depths in a water-filled phantom. The Variation Index (VI), (average modulus of %differences) was calculated. The more uniform the spread of values then the VI value is smaller. Table 2.

**Table 1 (abstract A37).** ᅟ

	Pb plate	LEHR collimator	Intrinsic
VI	0.58	4.35	3.1

Results were also confirmed with Simind Monte Carlo modelling.

**Table 2 (abstract A37).** ᅟ

	Pb plate	LEHR collimator	Intrinsic
VI	0.58	4.35	3.1

Results were also confirmed with Simind Monte Carlo modelling.

## A38 Whole body F-18-FDG-PET and tuberculosis: sensitivity compared to x-ray-CT

### H Huber^1^, I Stelzmüller^2^, R Wunn^3^, M Mandl^2^, F Fellner^3^, B Lamprecht^2^, M Gabriel^1^

#### ^1^Institut für Nuklearmedizin und Endokrinologie, AKH Linz/Kepler Universitätsklinikum, Austria; ^2^Abteilung für Lungenkrankheiten, AKH Linz/Kepler Universitätsklinikum, Austria; ^3^Zentrales Radiologie-Institut, AKH Linz/Kepler Universitätsklinikum, Austria

**Aim:** We did a retrospective survey of the results of F-18-PET-CTs and CTs performed at our hospital between 07/2008 and 08/2014 for suspected tuberculosis.

**Methods:** We included 197 patients (118 m, 79 f, age 53.7 ± 16.6 years at first visit); persons with prior malignant disease were excluded. They were scanned 50 min. past injection of (avg.) 300 MBq F-18-FDG from skull base to mid-femurs; these results were compared to diagnostic X-ray-CTs (including contrast enhancement where applicable) performed at the same visit or up to 4 weeks prior to the FDG-PET. We used microbiological findings as well as clinical and further (PET/)CT results to establish the diagnose independently.

**Results:** 83 patients showed no relevant uptake. In 21 we suspected a malignoma, which was proven in 9 cases by surgery and histology. The rest (105 patients) showed conspicuous focal uptake (SUV > 2.5), which was interpreted by us or after exclusion of malignant disease as inflammatory (tuberculosis, sarcoidosis and non-granulomatous). In 35 cases (18 m, 17 f, 45.4 ± 18 y) we do not only have proof of tuberculotic disease other than by FDG-PET, but also ≥ 1 follow-up PET/CTs.

We stratified our results according to involved systems: lung and pleura / LN intrathor. / LN extrathor. / bones / visceral organs / other soft tissue. 1 patient remained without FDG-uptake, 15 showed 1 involved system, 13 had lesions in 2, 1 in 3 and 5 in 4. The figures extracted from Xray-CT alone are 12/0 (incl. one false negative also with FDG), 13/1, 9/2, 1/3, none with 4 or more involved systems.

The best match was found in lung and pleural involvement: 15 patients were positive with both modalities (albeit including 2 discrepancies); 16 patients with mediastinal lymph node disease were posotve according to FDG, but only 6 were morphologically positive, too. 5/15 cases of extrathoracic LN, 1/7 with PET+ parenchymatous organs were radiologically suspect, as were 3/9 with bone lesions.

**Conclusion:** Whole body F-18-FDG-PET/CT enhances the quality of results in tuberculotic disease dramatically over X-ray methods alone. Especially extrathoracic disease involvement is by far better documented by molecular imaging, with probably considerable impact on treatment.

## A39 Emerging role 18F-FDG PET-CT in the diagnosis and follow-up of the infection in heartware ventricular assist system (HVAD)

### MC Fornito^1^, G Leonardi^2^

#### ^1^Nuclear Medicine Department and PET/CT center – A.R.N.A.S “Garibaldi – Nesima”, Via Palermo 636, 95122 Catania, Italy; ^2^Heart-Failure Department – Azienda Ospedaliera Universitaria “Policlinico- Vittorio Emanuele” Catania, Italy

**Aim:** A recent literature review has demonstrated that 18F-FDG PET/CT is becoming a very useful diagnostic tool in primary or material infection. In this study we evaluate the presence of infection in patient whit a mechanical heart detected by 18F-FDG PET/CT.

**Methods:** A 38 years-old male patient with a severe heart failure due to Becker’s muscular dystrophy underwent HeartWare ventricular assist system (HVAD) implant in 2012 as a bridge heart transplantation. Three years later, he complains of abdominal pain along the percutaneous driveline with associated infection local symptoms and abnormal biological parameters (VES and PCR). As conventional imaging (Ultrasonography, CT) and 99mTc-WBC scan were inconclusive, the heart-surgeons proposed to perform an 18F-FDG PET/CT to evaluate the extention of active infection process, on the basis of studies showing that cells involved in infection and inflammation, especially neutrophils and the monocyte/macrophage family, are able to express high levels of glucose transporters, especially GLUT1 and GLUT3, and hexokinase activity (in according to european guidelines). Patient attended a carbohydrates-free diet 48 hours before the exam, to switch miocardical glycolitc metabolism to free fatty acid. He fasted for at least 6 hours before 18F-FDG administration (blood glucose levels 97 mg/dl). Study was performed using an integrated PET-CT device (Philips Medical System) at 60 minutes from administration of radiotracer intravenously (323 MBq). CT was acquired from the base of the skull to the thighs (120 kV, 50 mA) and used for anatomical localization and attenuation correction of 3D-PET emission data. A second acquisition after 180 minutes was acquired to evaluate the SUV value variation.

**Results:** PET/CT scan showed linear area of FDG accumulation along the driveline, extending intracutaneous right abdomen region to the intracardiac implantation; the uptake has increased significantly in the late acquisition (∆SUV 25 %). Therefore PET-CT images were not able to evaluate the distribution of the radiotracer in miocardical wall near cardiac pump for the interference generated by metallic components on CT recostruction. Non attenuation corrected images confirmed the pathological uptake in driveline and exclude abnormal accumulation of radiotracer in the cardiac pump. In according to PET-CT scan the heart-surgeons decided to perform a cutaneous biopsy in order to identify microorganism for adapted antibiotic treatment. A new PET-CT will be performed as follow-up.

**Conclusion:** 18F-FDG PET-CT could be an appropriate imaging modality for management infection diseases in Patient with HVAD.

**Consent**

Written informed consent was obtained from the patient for publication of this abstract and any accompanying images. A copy of the written consent is available for review by the Editor of this journal.

**References**

1. Jongho K, Erika D, Wengen C., Vasken D. FDG PET/CT Imaging for LVAD Associated Infections . J Am Coll Cardiol Img. 2014;7(8):839–842.

## A40 Validation of Poisson resampling software

### WH Thomson, J O’Brien, G James

#### Physics and Nuclear Medicine Department, City Hospital, Birmingham, UK

**Aim:** ‘Poisson-resampling' software modifies images to represent reduced time/activity. Such software is now being made available by companies and allows for retrospective studies of changes to image quality with reducing time or activity. Previous testing of the software has relied on checking acquired floods. However these may have non-uniformities and do not include very low counts/pixel. Checking the performance at very low counts/pixel is particularly relevant for nuclear medicine studies e.g. bone scans where approximately 50 % of pixels are 1–10 counts/pixel. We have developed appropriate statistical tests for Poisson resampling software.

**Methods:** Our alternative analysis used simulated uniform images created with the same count/pixel (i.e. 'noiseless floods'). This allowed us to statistically test the Poisson resampling software on pixel values down to one count/pixel. Since the images are noiseless, i.e. without the standard Poisson noise of nuclear medicine images, statistical testing was different. Mathematically it can be shown that the Poisson software should generate a binomial (Gaussian) distribution with mean sqrt(f*(1-f)*M)), f = Poisson fraction, M = count/pixel of original image. Resampled data was tested using χ2 analysis.

**Results:** The Poisson resampling software showed excellent agreement with the expected binomial distributions for all values examined, including the lowest of 1 count / pixel (p > 0.1 for all cases).

**Conclusion:** This independent validation technique proves Poisson resampling software correctly generates expected noise characteristics even down to 1 count/pixel. This is reassuring given the increasing applications of such software, and indicates that the Poisson software can reliably be used in studies to examine the effects of reduced time or activity.

## A41 Protection of PET nuclear medicine personnel: problems in satisfying dose limit requirements

### J Hudzietzová^1^, J Sabol^2^, M Fülöp^3^

#### ^1^Faculty of Biomedical Engineering, CTU, Prague, Czech Republic; ^2^Faculty of Safety Management, PACR, Prague, Czech Republic; ^3^Faculty of Public Health, SMU, Bratislava, Slovak Republic

**Aim:** The paper is to discuss some current specific difficulties in assessing the radiation exposure received by workers handling radiopharmaceuticals at nuclear medicine clinics or departments engaged in the PET/CT examinations. This staff is daily coming into contact with unsealed radioactive material which results in its whole-body exposure as well as in the exposure of extremities, especially the skin of hands.

**Methods:** The present situation in radiation exposures of extremities of radiation workers has been summarized and the specific problems in monitoring the skin dose identified. The equivalent dose to the skin of hands of workers at selected nuclear medicine departments in the Czech Republic have been measured and compared against relevant dose limits.

**Results:** The distribution of the dose on the surface of fingers at specified positions monitored by TLDs is presented. The relation between the readings of finger dosimeters and the maximum skin exposure was analysed and interpreted. The preliminary results have shown that about 5 % of workers handling F-18 labelled radiopharmaceuticals may exceed the current dose limit for the skin.

**Conclusion:** The results of this study are consistent with our previous measurements [1] and other recently published communications (e.g. [2]) which also concluded that some nuclear medicine workers may not comply with the regulatory requirements relevant to the skin exposure. Due attention should be paid to a more realistic assessment of the skin doses taking into account the correction factors between the reading of ring dosimeters and the maximum skin dose as well as the possible impact of the radioactive contamination of gloves.

**References**

[1] Hudzietzová, J. et al. Assessment of the local exposure of skin on hands of nuclear medicine workers handling F-18 radiopharmaceuticals: Preliminary Czech study. Paper accepted for the publication in Radiation Protection Dosimetry (September 2015).

[2] ORAMED Final Report Summary: Optimization of radiation protection of medical staff. http://cordis.europa.eu/result/rcn/55187_en.html (retrieved on 2015-10-25).

